# Genome-wide interacting effects of sucrose and herbicide-mediated stress in *Arabidopsis thaliana*: novel insights into atrazine toxicity and sucrose-induced tolerance

**DOI:** 10.1186/1471-2164-8-450

**Published:** 2007-12-05

**Authors:** Fanny Ramel, Cécile Sulmon, Francisco Cabello-Hurtado, Ludivine Taconnat, Marie-Laure Martin-Magniette, Jean-Pierre Renou, Abdelhak El Amrani, Ivan Couée, Gwenola Gouesbet

**Affiliations:** 1 Centre National de la Recherche Scientifique, Université de Rennes 1, UMR 6553 ECOBIO, Campus de Beaulieu, bâtiment 14A, F-35042 Rennes Cedex, France; 2 UMR INRA 1165-CNRS 8114-UEVE, Unité de Recherche en Génomique Végétale (URGV), 2, rue Gaston Crémieux, CP5708, F-91057 Evry Cedex, France; 3 UMR AgroParisTech-INRA, Mathématique et Informatique Appliquées 518, F-75231 Paris Cedex 05, France

## Abstract

**Background:**

Soluble sugars, which play a central role in plant structure and metabolism, are also involved in the responses to a number of stresses, and act as metabolite signalling molecules that activate specific or hormone-crosstalk transduction pathways. The different roles of exogenous sucrose in the tolerance of *Arabidopsis thaliana *plantlets to the herbicide atrazine and oxidative stress were studied by a transcriptomic approach using CATMA arrays.

**Results:**

Parallel situations of xenobiotic stress and sucrose-induced tolerance in the presence of atrazine, of sucrose, and of sucrose plus atrazine were compared. These approaches revealed that atrazine affected gene expression and therefore seedling physiology at a much larger scale than previously described, with potential impairment of protein translation and of reactive-oxygen-species (ROS) defence mechanisms. Correlatively, sucrose-induced protection against atrazine injury was associated with important modifications of gene expression related to ROS defence mechanisms and repair mechanisms. These protection-related changes of gene expression did not result only from the effects of sucrose itself, but from combined effects of sucrose and atrazine, thus strongly suggesting important interactions of sucrose and xenobiotic signalling or of sucrose and ROS signalling.

**Conclusion:**

These interactions resulted in characteristic differential expression of gene families such as ascorbate peroxidases, glutathione-S-transferases and cytochrome P450s, and in the early induction of an original set of transcription factors. These genes used as molecular markers will eventually be of great importance in the context of xenobiotic tolerance and phytoremediation.

## Background

Different classes of herbicide act on plants through direct induction of oxidative injury. Herbicides of the triazine, phenolic and urea families, which bind to the D1 protein, inhibit photosystem II (PSII), and block electron transfer to the plastoquinone pool [1], thus causing the production of triplet chlorophyll and singlet oxygen (^1^O_2_). In cyanobacterial cells, ^1^O_2 _has been shown to cause direct photodamage to PSII and D1 protein and to prevent PSII repair by suppressing elongation of D1 protein [2]. Furthermore, ^1^O_2 _may generate other reactive oxygen species (ROS), such as hydroxyl radical (HO^•^) [3], and probably superoxide anion (O_2_^•-^) [4]. ^1^O_2 _can also act as a signalling molecule inducing stress and necrotic responses in Arabidopsis [5]. The lethal effects of PSII inhibitors can thus be ascribed to ROS injury rather than to nutritional stress and carbon starvation [1,6].

Exogenous treatment with sucrose, and to a lesser extent with glucose, was found to confer to Arabidopsis plantlets a very high level of tolerance to the triazine herbicide atrazine [7-9]. Sugar-treated plants were able to maintain PSII activity and phototrophic growth in the presence of atrazine concentrations, up to 40 μM, that are otherwise lethal, in the absence of sugar treatment. Moreover, sucrose-protected atrazine-treated Arabidopsis plantlets maintained active growth and oxygen evolution [7,8], thus suggesting that other mechanisms than phototrophic-photoheterotrophic transitions may be involved in sucrose-based protection against atrazine and ROS injury. Since notable differences of protection were conferred by glucose and sucrose for the same supply of carbon equivalents [7,8], we reasoned that protection also involved other physiological effects than metabolic feeding to energy and anti-oxidative pathways.

The demonstration that sugars acted as regulators of gene expression in plants [10,11] has led to the characterisation of a growing number of sugar-regulated genes. Thus, glucose or sucrose treatment in the absence of abiotic stress usually represses photosynthesis-related genes in plants [12] and in cyanobacterial cells [13]. This is the case for *psbA *mRNA and D1 protein accumulation in higher plants [7,12]. In the cyanobacterium *Synechocystis*, glucose feeding depresses the steady-state mRNA levels of PSII genes [13] and, under dark conditions, induces the destabilisation of *psbA *transcripts [14]. Surprisingly, sucrose treatment of Arabidopsis plantlets in the presence of atrazine results in markedly enhanced accumulation of *psbA *mRNA and D1 protein, which could be interpreted as derepression of sugar-induced repression of photosynthesis-related genes [7]. Moreover, application of ROS, especially H_2_O_2_, or changes of the glutathione redox state in the dark enhance *psbA *gene expression, which may thus help replenish D1 protein under conditions of oxidative stress [15]. Given that atrazine treatment itself had negative effects on D1 protein levels, the observed derepression in the presence of sucrose and atrazine [7] was therefore likely to result from interactions between sugar and oxidative stimuli. On the other hand, typical markers of ROS response have been shown to respond to interacting sugar and oxidative cues. Thus, Sulmon *et al*. [8] showed that, during sucrose-induced protection against atrazine treatment, *FSD1 *(encoding a chloroplastic Fe-superoxide dismutase) gene expression, which was slightly increased by sugar treatment *per se *and did not respond to atrazine treatment *per se*, was greatly enhanced in the presence of both sucrose and atrazine.

However, the extent of these interacting effects is not known. As outlined by Thum *et al*. [16] in their study of light and carbon signalling, the general picture of how interactions between sucrose and xenobiotic affect gene regulation must be gleaned from large-scale transcriptomic studies. In order to characterize these interactions and to obtain further insight into the roles of exogenous sugars in the tolerance to herbicides and oxidative stress, a CATMA whole Arabidopsis genome array [17] approach was undertaken. The microarray analysis was used to characterise the responses of Arabidopsis plantlets treated for 24 h in the presence of atrazine alone, sucrose alone, and sucrose plus atrazine and was complemented with a time-course study of a subset of genes using quantitative real-time reverse transcription-polymerase chain reaction (qRT-PCR). This genome-wide approach revealed that the primary effects of atrazine affected seedling physiology at a much larger scale than usually described in the literature, with the potential impairment of protein translation and of ROS defence mechanism, and that, correlatively, sucrose conferred atrazine protection through important modifications of transcript levels, not only as an effect of sucrose itself on stress response genes, but also as a result of sucrose and atrazine interactions that revealed the induction of novel stress defence genes. Finally, since sucrose application, which enhances xenobiotic tolerance, accumulation in plants, and consecutively decontamination of surrounding soil, appears to be potentially useful for phytoremediation [9,18,19], characterization of gene markers related to xenobiotic protection will be important to analyse phytoremediation properties in the field.

## Results

### Physiological effects of atrazine and sucrose treatments

In order to obtain insight into sucrose-induced atrazine tolerance, to characterize specific and beneficial effects of sucrose and to establish an analysis of gene functions under tolerance or stress conditions, the first step was to determine a treatment yielding plantlets at an early stage of injury, and presenting effects on gene transcription prior to advanced and visible effects of cell death. Thus, at 24 h of transfer, atrazine-treated plantlets could be compared to mannitol-treated, sucrose-treated, and sucrose plus atrazine-treated plantlets which had not yet undergone significant developmental changes resulting from the treatment and were therefore in a comparable physiological state (Additional file 1). In order to study the same developmental stage of plantlets, Arabidopsis plantlets were grown on Murashige and Skoog agar medium and transferred at the 1.02 development stage [20] to Murashige and Skoog agar medium supplemented with mannitol (80 mM) as osmotic control (M), mannitol (80 mM) plus atrazine (10 μM) as herbicide treatment (MA), sucrose (80 mM) as sugar effect control (S) and sucrose (80 mM) plus atrazine (10 μM) as protective treatment (SA). MA treatment induced complete bleaching of plantlets after 6 to 7 days of stress application, thus leading to seedling death within 8 days (Additional file 1). In contrast SA treatments allowed plantlets to maintain growth and development beyond 8 days of transfer. The herbicide treatment produced root growth inhibition upon 24 h of transfer, while the other conditions resulted in recovery of root growth within 2 days of treatment. Chlorophyll and carotenoid contents were identical for all treatments upon 24 h of transfer (Additional files 2 and 3). Pigment contents were maintained between 4 and 8 days of SA treatment whereas these values decreased between 4 and 8 days of MA treatment. This difference between MA and SA treatments resulted in complete death of MA plantlets and in maintenance of growth and development in SA plantlets. This was correlated with measurements of photosystem II efficiency (Fv/Fm), which stayed unchanged for all treatments after 24 h of treatment (Additional file 4). After 8 days of SA treatment, PSII still showed significant efficiency, while no PSII efficiency was detected for the herbicide treatment.

Given that MA treatment induced significant injuries on shoot physiology and root growth from 24 h to 8 days, thus resulting in various secondary effects related to injury and bleaching, we chose to study plantlets at 24 h of treatment where primary effects of atrazine exposure could be analysed.

### Effects of atrazine and sucrose on global gene expression

Mannitol treatment (M) was chosen as the control for all comparisons in order to filter osmotic-responsive genes (Additional file 5). Differentially expressed genes, i.e. showing at least one *P*-value ≤ 0.05 after Bonferroni correction, in MA/M, SA/M or S/M comparisons were selected. Among the 24 576 gene-specific tags, corresponding to 22 089 genes plus 516 chloroplastic and mitochondrial probes, 5304 probes (24% of represented genes) were significantly differentially expressed in at least one of the 3 comparisons with only 44 belonging to the mitochondrial genome and 41 to the chloroplastic genome.

**Figure 1:**
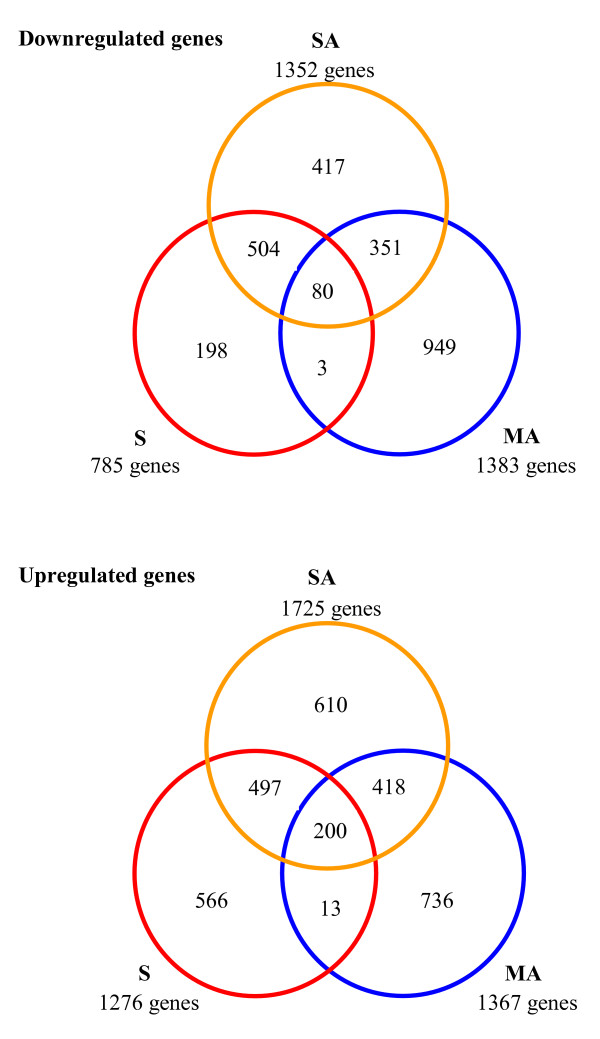
Venn diagrams showing the distribution and overlap of transcript modifications in Arabidopsis plantlets transferred on sucrose medium (S), sucrose plus atrazine medium (SA) and mannitol plus atrazine medium (MA). A statistical cut off (*P*-value ≤ 0.05 after Bonferroni correction) was used to determine which genes were significantly differentially expressed in comparison with the mannitol treatment.

In order to define specificity and crosstalk among the transcriptomic responses for the studied treatments, we analysed overlapping of downregulated and upregulated genes for S, SA and MA treatments (Figure 1). The data set clearly demonstrated that the stress condition (MA) was responsible for downregulation of a large quantity of transcripts: 949 specifically atrazine-repressed genes compared to 417 and 198 genes specifically repressed by the sucrose-atrazine combination and sucrose alone, respectively. The low number of genes commonly repressed (3 genes) or induced (13 genes) by sucrose alone or by atrazine alone and unaffected by SA treatment revealed the important differences of the gene expression modifications between the two treatments. Atrazine-tolerant plantlets (SA) presented a larger number of upregulated genes (1725 genes) than plantlets under the other conditions (1276 and 1367 genes for, respectively, S and MA treatments). Given that the SA treatment shared one parameter with the other treatments, the large number of genes specifically induced (610 genes) or repressed (417 genes) in the simultaneous presence of atrazine and sucrose strongly indicated a combined effect of the two factors.

The normalized log_2_(ratio) generated by the statistical analysis was used to describe the extent of transcriptional change between the MA/M, SA/M and S/M comparisons. In order to focus the study on highly repressed or induced transcripts, genes whose log_2_(ratio) was greater than 1.585 or lower than -1.585 (corresponding to 3-fold change) in at least one comparison were considered as highly-responsive. These criteria gave a total of 810 highly-responsive genes available in Additional file 6. Among these selected genes, 191 and 164 loci were found to be, respectively, upregulated and downregulated by atrazine treatment. Sucrose alone was responsible for increase of expression of 147 genes and decrease of expression of 113 genes. Under tolerance conditions, atrazine and sucrose generated induction of 321 genes and repression of 94 genes. Contrary to other treatments, sucrose-treated atrazine-tolerant plantlets therefore showed strong increase in upregulated genes correlated with a decrease in downregulated genes, thus suggesting induction of specific protective mechanisms.

The expression profiles of 8 genes, belonging to various functional categories and showing various expression patterns in the microarray, were analysed by qRT-PCR under the same conditions as those of the microarray experiment (Additional file 7). In all cases, due to the normalization steps, the fold changes obtained with the qRT-PCR were higher than those on the microarray, but gave comparable expression profiles relatively to the different treatments.

### Identification of protection-related functional categories

**Figure 2:**
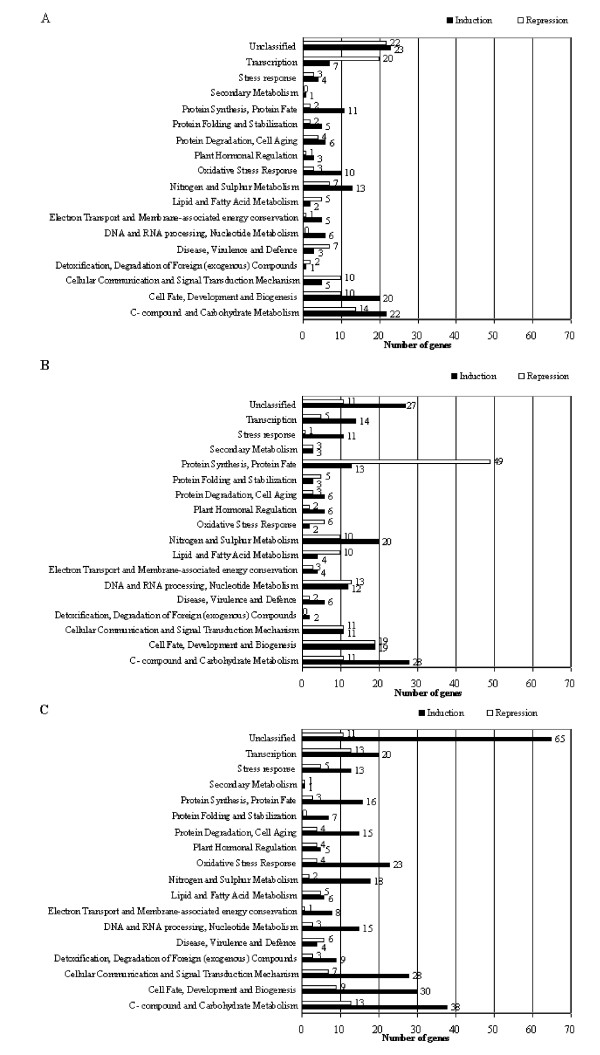
Overview of induced and repressed biological processes after sucrose, atrazine or sucrose plus atrazine treatment of Arabidopsis plantlets. Genes were classified according to annotations from the Munich Information Centre for Protein Sequence database (MIPS) [21]. Numbers of genes identified are indicated on the horizontal axis. A, effects of sucrose on functional distribution; B, effects of atrazine on functional distribution; C, effects of sucrose and atrazine on functional distribution.

In order to investigate biological processes involved, transcripts showing 3-fold variations of expression were assigned to functional categories based on the Munich Information Centre for Protein Sequence database (MIPS) [21,22] (Figure 2A,B,C). Transcripts with no clearly ascertainable role were labelled as unclassified. The statistical significance of differences of repression and induction is described in Additional file 8.

The majority of sucrose-induced transcripts (Figure 2A) were involved in protein synthesis/protein fate, nitrogen and sulphur metabolism, DNA and RNA processing, cell fate, development and biogenesis, and C-compound and carbohydrate metabolism. Such effects of exogenous carbohydrates have been described in previous studies [10,16,23].

Figure 2B shows direct or indirect modifications caused by atrazine alone. Induction of genes involved in stress response, detoxification and degradation of foreign compounds, disease virulence and defence was observed. Similar patterns of gene responses have been described in response to oxidative stress in Arabidopsis [5,24,25]. Genes involved in protein synthesis, protein fate were largely downregulated and represented about 30% of repressed genes, whereas expression of genes related to protein degradation/cell aging was slightly increased. It must be noted that atrazine induction of genes involved in oxidative stress response (2 induced genes) was significantly low in comparison with the corresponding sucrose (10 induced genes) and sucrose plus atrazine (23 induced genes) inductions. The irreversible cellular injury caused by atrazine may thus be related to modifications of oxidative stress responses and of protein dynamics besides direct molecular damage of oxidative stress leading to cell death which has been widely described [25,26].

The concurrent presence of atrazine and sucrose was associated with large induction of gene expression particularly related to cellular communication and signal transduction mechanism, detoxification and degradation of foreign compounds, oxidative stress responses, and protein degradation/cell aging (Figure 2C). Genes linked to detoxification and degradation of foreign compounds, oxidative stress response and cellular communication and signal transduction mechanism were thus largely induced in comparison with the atrazine alone or sucrose alone treatments, thus suggesting strong synergic effects of sucrose and atrazine on protection pathways. Induction of DNA/RNA processing, nucleotide metabolism (15 induced genes) and transcription (20 induced genes) categories by sucrose plus atrazine was significantly higher than that by sucrose alone, and also contrasted with the significant repression of these categories in the atrazine alone treatment.

In order to distinguish induction or repression resulting from the effect of atrazine and sucrose combination, we used the comparison of mannitol-atrazine treatment against sucrose-atrazine treatment (MA/SA) for detecting expression variations resulting only from the effect of atrazine, and for expression variations resulting only from the effect of sucrose, we used the comparison of sucrose treatment and sucrose plus atrazine treatment (S/SA). Transcripts presenting a *P*-value > 0.05 (not differentially expressed) in these comparisons were subtracted from the list of 810 highly-responsive genes, and then all the genes whose expression was not modified by addition of atrazine in the presence of sucrose (not differentially expressed in the S/SA comparison) and which were constant between the stress condition and the tolerance condition (not differentially expressed in the MA/SA comparison) were also removed. Among the 410 resulting genes, thus largely controlled by the combination of atrazine and sucrose, only 16 genes were strongly downregulated (more than 3-fold) against 136 genes strongly upregulated (more than 3-fold). These data, which corroborated the Venn diagram distribution (Figure 1), reinforced the idea of induction of specific stress tolerance mechanisms against the harmful effects of the atrazine treatment.

**Figure 3:**
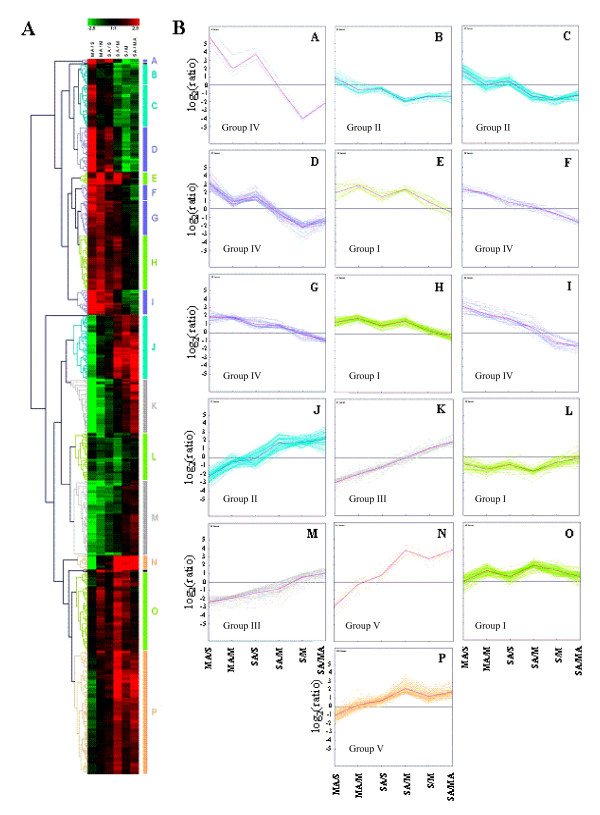
Schematic representation of the expression pattern of the 810 highly-responsive genes in the six comparisons of the 24 h endpoint treatment. (A) Average linkage hierarchical clustering, where each horizontal line displays the expression data for one gene. The colours red or green indicate respectively up- and downregulation. (B) Representation of the 16 clusters associated to the hierarchical clustering.

In order to identify relationships between sucrose- and atrazine-responsive genes, the 810 highly-responsive genes previously selected were subjected to hierarchical clustering (LHC) using Euclidian distance for the similarity distance and the average linkage clustering for the linkage rule (Figure 3). This analysis classified genes into 16 clusters (A to P) according to their expression profiles for the 6 comparisons (MA/S, MA/M, SA/S, SA/M, S/M and SA/MA) described in Methods. These 16 clusters were organised into 5 groups representative of specific patterns (Additional file 6). For group I (clusters E, H, L and O), the SA/MA comparison revealed no differential expression and was correlated to similar log_2_(ratio) for SA/M and MA/M comparisons with positive values for clusters E, H and O and negative for cluster L. Group I included genes with similar expression for atrazine treatment in the presence or absence of sucrose, thus corresponding to loci principally regulated by atrazine. In contrast, group II included genes with similar expression for sucrose or sucrose-atrazine treatment (clusters B, C and J), thus corresponding to genes principally regulated by sucrose. Indeed, these genes were not differentially expressed in the comparison SA/S and showed comparable log_2_(ratio) for the SA/M and S/M comparisons with positive log_2_(ratio) for cluster J and negative log_2_(ratio) for clusters B and C. Group III (clusters K and M) comprised genes whose expression was repressed by atrazine alone (MA/M comparison) and induced by sugar alone (S/M comparison) and for which the atrazine-sucrose combination (SA/M comparison) averaged expression values by balancing negative effects of atrazine and positive effects of sucrose. Group IV (clusters A, D, F, G and I) presented a similar behaviour to that of group III but with opposite effects of sucrose alone and atrazine alone on gene expression. Finally group V (cluster N and P) exhibited high induction of expression by combined sucrose-atrazine treatment, which was not produced by additional effects of sucrose alone and atrazine alone. Transcripts from that group seemed to be highly-induced specifically by sucrose-atrazine combination.

### Characterization of atrazine xenobiotic and oxidative effects: evidence for deleterious effects on gene regulation

Genes showing large induction or repression of expression for atrazine treatment were principally ascribed to groups I, III and IV (Figure 3). In a context of ROS injury, atrazine induced some potentially adaptative responses (Table 1), such as induction of an ABC transporter (At1g70610, group I) and a multidrug and toxic compound extrusion family protein MATE (At1g33110, group I) involved in detoxification of glutathione-conjugated xenobiotics and their transport from cytoplasm to vacuole [27]. Moreover, atrazine upregulated the glutathione-mediated system of methylglyoxal detoxification, which involves glyoxalase I (At1g80160, group IV) and putative glyoxalase II (At1g53580, group I) [28].

**Table 1:** Induction by atrazine of genes involved in xenobiotic and oxidative stress response

		log_2_(ratio)		
		
		Treatment comparison		
Accession number	Gene description	MA/M	S/M	SA/M
At1g06570	4-hydroxyphenylpyruvate dioxygenase (PDS1)	3.19	-0.76	2.12
At1g33110	MATE efflux family protein	2.17	nde	1.88
At1g53580	Hydroxyacylglutathione hydrolase, putative/glyoxalase II putative	1.75	nde	1.06
At1g70610	ABC transporter (TAP1)	2.20	nde	1.38
At1g80160	Glyoxalase I family protein	2.03	-1.81	nde

nde: not differentially expressed, genes with a Bonferroni *P*-values higher than 5% were considered as being not differentially expressed as described in Lurin *et al*. [75].

Atrazine also upregulated *PDS1 *transcripts (At1g06570, group IV), encoding a 4-hydroxyphenylpyruvate dioxygenase involved in biosynthesis of plastoquinones, tocopherols and carotenoids [29], which are essential elements of photosynthetic electron transport chain and of antioxidative systems. The antioxidant properties of tocopherols arise from their ability to scavenge lipid peroxy radicals before they react with lipid substrates. Carotenoids play a key role in protection of PSII against photoinhibition, since they are able to quench ^1^O_2 _responsible for photooxidative damage [30].

Atrazine stress thus induced protective mechanisms that seemed to be partial and inefficient inasmuch as they did not eventually prevent plant death. Moreover, analysis of atrazine treatment showed repression of numerous genes potentially important for xenobiotic or oxidative response (Table 2), such as (i) the gene encoding the antioxidant enzyme L-ascorbate peroxidase 1 APX1 (At1g07890, group III) which belongs to the ascorbate-glutathione cycle and can scavenge oxygen free radicals, thereby minimising injury caused by oxidative stress [31], (ii) glutathione S-transferases (GSTs) AtGSTU20 (At1g78370, group III) and AtGSTF11 (At3g03190, group III), which are detoxifying enzymes present in all aerobic organisms and catalyse conjugation of glutathione (GSH) with a variety of electrophilic compounds, including triazines. GST isoforms presented different patterns of gene expression: some of them were largely repressed by atrazine whereas the concomitant presence of herbicide and sucrose resulted in a return to basal level observed in the presence of sucrose alone. This was the case for AtGSTF11, which has been shown to be active both as a GST and as a glutathione peroxidase [32].

**Table 2:** Repression by atrazine of genes involved in xenobiotic and oxidative stress response

		log_2_(ratio)		
		
		Treatment comparison		
Accession number	Gene description	MA/M	S/M	SA/M
At1g07890	L-ascorbate peroxidase 1, cytosolic (APX1)	-1.82	nde	nde
At1g78370	Glutathione S-transferase, putative (AtGSTU20)	-3.72	0.82	-0.92
At2g34490	Cytochrome P450 family protein (CYP710A2)	-2.96	nde	nde
At2g47470	PDIL, thioredoxin family protein	-1.74	nde	nde
At3g03190	Glutathione S-transferase, putative (AtGSTF11)	-1.80	nde	-0.85
At4g04830	MrsB5, methionine sulfoxide reductase domain-containing protein/SeIR domain-containing protein	-2.06	nde	nde
At5g42650	Allene oxide synthase (AOS)	-2.76	nde	-2.55

nde: not differentially expressed, genes with a Bonferroni *P*-values higher than 5% were considered as being not differentially expressed as described in Lurin *et al*. [75].

The cytosolic methionine sulfoxide reductase B5 MrsB5 (At4g04830, group I) was also downregulated by addition of atrazine. ROS-mediated oxidation of methionine into methionine sulfoxide (MetO) is a major component of oxidative damage to proteins. Methionine sulfoxide reductase (Msr) systems reduce MetO to protect plant cells from cytotoxic effects and thereby prevent excessive accumulation of oxidized proteins and premature death during defence mechanisms [33]. Vieira Dos Santos *et al*. [34] showed that MsrB protein amount increased after photooxidative stress, thus suggesting a role in the protection of cells against oxidative damage. Vignols *et al*. [35] demonstrated that thioredoxins (TRX) directly interact with Msr *in vivo *and could then act as electron donor to Msr proteins, thus suggesting the existence of a linked antioxidative mechanism. However, our results showed that transcripts of At2g47470 (group III) encoding a protein disulfide isomerase-like (PDIL) protein, a member of a multigene family within the TRX superfamily, were repressed by atrazine as observed for *MsrB5*. TRX are involved in the regulation of cellular redox balance by reducing disulfide bridges, and in a large panel of mechanisms like defence, development and antioxidative responses [36]. Meyer *et al*. [36] suggested a crosstalk between TRX and glutaredoxins, thus leading to a potential link between TRX, glutathione and glutathione reductase. One of the proposed TRX targets in Arabidopsis is the APX1 protein [36], whose transcripts were repressed in the presence of atrazine. In our analysis, all of these transcripts involved in xenobiotic and oxidative stress defence belonged principally to group III (Figure 3) and were downregulated by atrazine, thus probably preventing their protective role.

All of these results strongly suggested a lack of an efficient anti-oxidative response in the presence of atrazine. Moreover, atrazine treatment was associated with strong repression of genes involved in nucleic acid and protein dynamics (Additional file 9). Indeed, among the 810 highly-responsive genes selected for data analysis, 81 belonged to the protein dynamics category (Protein Synthesis/Modification or Degradation) and 60% of these genes were found to be downregulated by the herbicide. Among the 36 genes involved in nucleic acids dynamics, 35% of transcripts were repressed by atrazine, whereas the sucrose/atrazine treatment induced about 40% of them.

Other potentially negative effects of atrazine on plant cell physiology (Table 3) involved repression of Tubulin TUA4 (At1g04820, group III) transcription, or downregulation of *EXPB3 *(At4g28250, group III) and *EXP15 *(At2g03090, group III) transcripts, which are involved in cell elongation. The mitochondrial NADH-cytochrome-b5 reductase (At5g20080, group III) involved in electron transport through its oxidoreductase activity was negatively controlled by atrazine, which may thus hamper mitochondrial respiration. Finally, the FKBP15-2 immunophilin (At4g25340, group III), which has peptidyl prolyl cis/trans isomerase (PPIase) activity involved in protein folding processes, was repressed by atrazine, which may lead to inefficient protein conformations.

**Table 3:** Selected atrazine-regulated genes that may be involved in atrazine injury

		log_2_(ratio)		
		
		Treatment comparison		
Accession number	Gene description	MA/M	S/M	SA/M
At1g03090	3-methylcrotonyl-CoA carboxylase 1 (MCCA)	1.87	-1.66	nde
At1g04820	Tubulin alpha-2/alpha-4 chain (TUA4)	-2.33	0.76	nde
At1g66330	Senescence-associated family protein	2.10	nde	nde
At2g03090	Expansin, putative (EXP15)	-2.01	nde	nde
At2g22980	Serine carboxypeptidase S10 family protein	1.16	-2.02	nde
At3g45140	Lipoxygenase (LOX2)	-2.94	nde	-1.89
At3g45300	isovaleryl-CoA-dehydrogenase (IVD)	2.79	nde	1.34
At4g16190	Cysteine proteinase, putative	2.21	nde	1.04
At4g17040	ATP-dependent Clp protease proteolytic subunit, putative	1.68	0.96	1.15
At4g25340	FKBP15-2 immunophilin/FKBP-type peptidyl-prolyl cis-trans isomerase-related	-1.73	nde	nde
At4g28250	Beta-expansin, putative (EXPB3)	-2.21	1.10	nde
At5g20080	NADH-cytochrome b5 reductase, putative	-1.70	0.98	nde
At5g35630	Glutamine synthetase (GS2)	1.90	0.92	1.02

nde: not differentially expressed, genes with a Bonferroni *P*-values higher than 5% were considered as being not differentially expressed as described in Lurin *et al*. [75].

Moreover, Table 3 shows that, in presence of atrazine, an ATP-dependent Clp protease (At4g17040, group I) was induced. Clp proteases in chloroplasts degrade misfolded or unassembled proteins in an ATP-dependent manner, in relation to the activity of molecular chaperones, in order to target specific polypeptide substrates and avoid inadvertent degradation of others [37]. Accumulation of transcripts of Clp protease is upregulated during several stresses [38]. Moreover, genes involved in amino acid catabolism, such as At1g03090 (group IV) encoding the 3-Methylcrotonyl-coenzyme A carboxylase (MCCase), At3g45300 (group IV) encoding a isovaleryl-CoA-dehydrogenase (IVD), and genes involved in nitrogen salvaging such as At5g35630 (group I) encoding glutamine synthetase GS2, were upregulated during atrazine stress. Their implication during protein degradation has been previously described [39-41]. This increase may reflect a situation of carbohydrate starvation [41,42]. However, other typical markers of carbohydrate starvation and autolysis regulation [42], such as the At3g48920 MYB transcription factor (TF), catalase 3 (At1g20620) and APG8 autophagy genes (At3g06420, At4g16520), did not respond to atrazine treatment, thus confirming that atrazine effects could not be primarily ascribed to carbohydrate starvation.

### Specific effects of combined sucrose plus atrazine treatment on tolerance-related gene regulation

Previous studies have already described the transcriptomics of sugar treatment in *Arabidopsis thaliana *[16,23,42,43]. The sucrose-alone control treatment was therefore compared in detail with previous studies in order to detect any anomaly or specificity of the sucrose-treated plantlets used in the present study. Our conditions of sucrose treatment resulted in modification of typical markers of carbohydrate responses in accordance with previous studies (Additional file 10) [10,23]. However, the observed gene expression modifications due to sucrose alone could not explain enhanced tolerance to atrazine, thus emphasising the importance of comparing sucrose-alone and sucrose plus atrazine treatments.

In group V, transcripts from cluster P (Figure 3) exhibited an expression pattern strongly suggesting specific regulation by sucrose-atrazine combination. Only a small number of genes in this group have been functionally characterised in previous studies (Table 4). Thus, the type 2C protein phosphatase (At4g31860, group V) has been described as ABA-inducible and involved in ABA signal transduction [44]. The *HEMA2 *gene (At1g09940, group V), which is involved in heme and chlorophyll biosynthesis, was also highly-induced by sucrose plus atrazine treatment. The pattern of expression of *HEMA2 *may thus be related to maintenance and repair of chloroplasts in the presence of atrazine and sucrose.

**Table 4:** Genes potentially involved in sucrose-induced atrazine tolerance

		log_2_(ratio)		
		
		Treatment comparison		
Accession number	Gene description	MA/M	S/M	SA/M
At1g09940	Glutamyl-tRNA reductase 2/GluTR (HEMA2)	nde	0.84	2.02
At1g69930	Glutathione S-transferase, putative (AtGSTU11)	nde	2.90	3.73
At2g18193	AAA-type ATPase family protein	-1.55	2.97	3.18
At2g34500	Cytochrome P450 family protein (CYP710A1)	nde	3.05	4.51
At2g41380	Embryo-abundant protein-related	nde	2.77	3.34
At3g22370	Alternative oxidase 1a, mitochondrial (AOX1A)	nde	2.76	3.27
At3g28210	Zinc finger (AN1-like) family protein (PMZ)	nde	2.75	3.19
At3g50930	AAA-type ATPase family protein	-1.19	2.71	3.20
At3g56710	SIB1, sigma factor binding protein	nde	nde	1.84
At4g11890	Protein kinase family protein	nde	1.64	3.82
At4g31860	Protein phosphatase 2C, putative/PP2C, putative	nde	nde	1.79
At4g33070	Pyruvate decarboxylase, putative (PDC)	nde	3.06	3.73
At5g02780	In2-1 protein, putative	nde	2.48	4.00
At5g09570	Expressed protein	nde	2.36	3.25
At5g54100	Band 7 family protein	nde	3.32	4.34
At5g54206	12-oxophytodienoate reductase-related	nde	2.72	3.67

nde: not differentially expressed, genes with a Bonferroni *P*-values higher than 5% were considered as being not differentially expressed as described in Lurin *et al*. [75].

Among group V, cluster N (Figure 3) showed further evidence for specific effects of sucrose and atrazine interactions (Table 4). This cluster contained genes encoding detoxifying enzymes like glutathione S-transferase AtGSTU11 (At1g69930, group V). Lacomme and Roby [45] demonstrated that expression of *AtGSTU11 *is induced in response to salicylic acid and methyl jasmonate and in response to avirulent pathogens causing a hypersensitive reaction. At5g02780 (group V) encodes an In2-1 protein that is induced in response to iron treatment [46]. However, analysis of At5g02780 sequence shows the presence of two conserved domains corresponding, respectively, to the N- and C-terminal domains of glutathione S-transferase, thus suggesting a role of this gene in detoxification mechanisms. A *PDC *gene (At4g33070, group V) encoding a pyruvate decarboxylase was induced by sucrose and to a much more higher level by the presence of sucrose plus atrazine. This At4g33070 locus is highly-expressed during anoxia [47], and exogenous sucrose, which enhances anoxia tolerance, correlatively increases At4g33070 transcript accumulation. Since induction of *PDC *genes has been described as a response to situations of abiotic stress leading to mitochondrial impairment [48], the present induction of the At4g33070 *PDC *gene may contribute to promote a back-up fermentative pathway that compensates mitochondrial impairment. Indeed, we have shown above that atrazine injury was associated with downregulation of a mitochondrial NADH-cytochrome-b5 reductase (At5g20080, group III), while a return to the basal level was observed under tolerance condition (Table 3). The mitochondrial AOX1A (At3g22370, group V) is known to use excess reductant capacity of the cytochrome c oxidase pathway, thus preventing ROS formation from an over-reduced ubiquinone pool [49]. *AOX1A *is induced by sucrose alone and much more by atrazine plus sucrose, which may thus increase potential antioxidative properties of this detoxifying enzyme through the glyoxylate pathway.

The sucrose plus atrazine treatment induced more than 22-fold the expression of a cytochrome P450 CYP710A1 (At2g34500, group V), responsible for a C22 desaturation reaction which produces stimasterol [50], which may thus contribute to maintain proper sterol composition of membranes and associated cell functions.

A number of genes involved in abiotic stress response were upregulated by the sucrose-atrazine combination (Figure 2C). Two genes encoding AAA Type ATPases (At3g50930, group V, At2g18193, group V) presented an important induction. This large protein family is involved, via chaperone-like activity, in numerous cellular activities including membrane fusion, proteolysis, DNA replication and repair, protein folding, and cytoskeletal regulation [51]. They were identified as highly upregulated after genotoxin application [52], and may thus contribute to defence/stress response or cell cycle control. Sucrose-atrazine treatment also activated a zinc finger TF (PMZ, At3g28210, group V) that is induced during senescence, pathogen infection and in the ozone-sensitive *vtc1 *mutant [53,54]. The embryo-abundant protein (At2g41380, group V) induced by sucrose-atrazine treatment has been described as induced by UV-B irradiation and ozone fumigation [55]. The At5g54100 (group V) and At5g09570 (group V) genes, of unknown function, have been shown to be induced by cesium treatment in roots [56].

### Differential expression of specific transcription factors during sucrose-induced atrazine tolerance

Plant acclimation to atrazine treatment may be controlled by a complex network of regulatory genes. It is estimated that approximately 5% of genes in the genome of eukaryotic organisms encode transcription factors (TFs) [57]. Among our selection of 810 genes corresponding to 3-fold variation, about 8% belonged to TF category. In order to focus on genes potentially involved in tolerance response, only TFs highly-regulated by the sucrose-atrazine treatment were selected (Table 5). Among these TFs, two WRKY TFs (At2g30250, group V, At5g13080, group V) and one AP2 domain-containing TF (At3g50260, group V) have already been considered as hallmarks for the general oxidative stress response responding to a signal related to oxidative cellular damage [24]. SIB1 (a chloroplastic Sigma Factor Binding Protein 1, At3g56710, group V) interacts with SibI, a protein with unknown function whose gene expression is tissue-specific, light-dependent, and developmentally-timed [58]. The bZIP TF AtbZIP60 (At1g42990, group V) was nearly exclusively induced by the sucrose-atrazine association. Iwata and Koizumi [59] demonstrated that AtbZIP60 is induced by the endoplasmic reticulum (ER) stress response (also called the unfolded protein response) and may regulate expression of ER chaperones. The zinc finger protein Zat12 (At5g59820, group V), which is also upregulated under tolerance condition, seems to play a central role in reactive oxygen and abiotic stress (cold, salinity) signalling [60]. Moreover, some results may suggest that Zat12 is part of a transcriptional or signal transduction complex required for *APX1 *expression, essential to prevent oxidative stress [31]. Another zinc finger TF (PMZ, At3g28210, group V), induced by sucrose alone and by sucrose-atrazine combination, is related to biotic stress response and to senescence [53,54]. The induction by sucrose-atrazine combination of AP2 TF (At2g40340, group V), a putative DRE2B (dehydration-responsive element-binding protein 2B), could also reflect its implication in stress response pathways. Nakashima *et al*. [61] had shown that *DRE2B *gene was induced by dehydration and high salinity that generate oxidative stress in plant. Other WRKY TFs, such as At2g23320 (group V), were strongly induced by sucrose-atrazine combination. Although these genes have not been specifically characterized, TFs of WRKY family are generally able to recognize various motifs present in promoters of many defence-related genes, and they are often upregulated in response to various stresses, including infections by pathogens, wounding, and oxidative stress conditions [62]. They regulate various aspects of plant development and cell death connected with ROS signalling events [62].

**Table 5:** Transcription factors potentially involved in sucrose-induced atrazine tolerance

		log_2_(ratio)		
		
		Treatment comparison		
Accession number	Gene description	MA/M	S/M	SA/M
At1g08050	Zinc finger (C3HC4-type RING finger) family protein	nde	0.87	1.77
At1g21910	AP2 domain-containing transcription factor family protein	nde	-0.96	-1.59
At1g42990	bZIP transcription factor family protein (AtbZIP60)	nde	0.78	1.96
At1g71520	AP2 domain-containing transcription factor, putative	nde	0.92	1.80
At2g23320	WRKY family transcription factor	nde	1.05	2.33
At2g30250	WRKY family transcription factor	0.75	1.14	2.10
At2g40340	AP2 domain-containing transcription factor, putative (DRE2B)	nde	2.21	3.28
At2g47890	Zinc finger (B-box type) family protein	1.19	1.37	3.05
At3g28210	Zinc finger (AN1-like) family protein (PMZ)	nde	2.75	3.19
At3g50260	AP2 domain-containing transcription factor, putative	nde	1.67	3.26
At3g56710	SIB1, sigma factor binding protein	nde	nde	1.84
At3g61630	AP2 domain-containing transcription factor, putative	nde	1.04	2.14
At5g13080	WRKY family transcription factor	0.79	2.00	2.89
At5g59820	Zinc finger (C2H2 type) family protein (Zat12)	nde	1.15	2.08

nde: not differentially expressed, genes with a Bonferroni *P*-values higher than 5% were considered as being not differentially expressed as described in Lurin *et al*. [75].

The potential involvement of these TFs was further investigated by the research of cis-acting regulatory elements in promoters of genes presenting high induction by the sucrose-atrazine combination (Group V, Figure 3). Identification of cis-acting regulatory elements was carried out using the AtcisDB database [63]. The most repeated and frequent motifs found were cis-acting regulatory elements corresponding to the WRKY, bZIP, MYB, and LFY TFs families (Additional file 11). Thus, the selected genes from group V (Figure 3), highly-responsive to sucrose-atrazine combination and potentially important for tolerance mechanisms, presented promoters with identified cis-acting regulatory elements corresponding to two of the TF families of Table 5, the WRKY and bZIP families.

In contrast, the MYB and LFY cis-acting regulatory elements did not correspond to TFs described in Table 5, thus suggesting that these MYB and LFY cis-elements may be regulated by TFs showing lower level of induction or expressed at earlier or later steps of the response. However, it was surprising that the identification of AP2 and Zinc finger TFs in Table 5 did not correspond to any cis-element in the promoters of group V genes. This may be due to the partial information contained in AtcisDB database, where the binding sites of the seven AP2 and Zinc finger genes of Table 5 are not described, thus suggesting that these specific binding sites are significantly different from consensus cis-elements.

### Time-course of induction of transcription factors during sucrose-dependent atrazine protection

Using Expression Angler with the AtGenExpress Stress Set [64], we selected, from Table 5, TFs which presented an expression pattern that was highly correlated (Pearson correlation coefficient between 0.78 and 0.96) with that of genes potentially related to sucrose-induced atrazine protection, such as *AOX1A *(At3g22370, group V) or AAA Type ATPases (At3g50930, group V). This analysis highlighted the potential importance of four genes encoding different families of TFs: Zinc finger protein Zat12 (At5g59820, group V), bZIP family protein AtbZIP60 (At1g42990, group V), Sigma factor binding protein SIB1 (At3g56710, group V) and AP2 domain-containing TF (At3g61630, group V).

**Figure 4:**
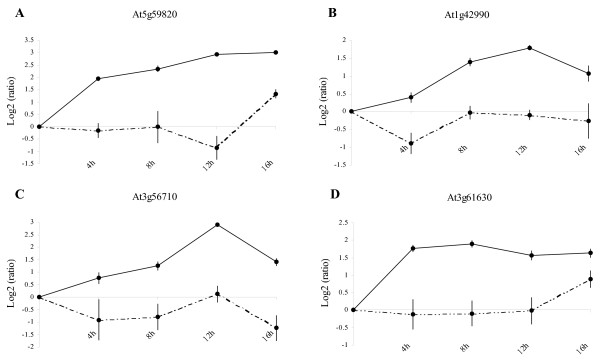
Time-course of transcription factor induction during sucrose-dependent atrazine protection. (A) Zinc finger protein Zat12 (At5g59820), (B) bZIP family protein AtbZIP60 (At1g42990), (C) Sigma factor binding protein SIB1 (At3g56710), (D) AP2 domain-containing TF (At3g61630). Plantlets were transferred during 4, 8, 12 or 16 h on mannitol as osmotic control, mannitol plus atrazine as herbicide treatment and sucrose plus atrazine as protective treatment. RNA from these plantlets was extracted and used for qRT-PCR analysis. The continuous line represents log_2_(ratio) of relative expression between sucrose plus atrazine treatment and the physiological control, while the discontinuous line represents log_2_(ratio) of relative expression between mannitol plus atrazine treatment and the physiological control.

The importance of these TFs in the development of sucrose-induced tolerance was assessed by a time-course study (Figure 4). The results of this time-course analysis confirmed results of the CATMA microarray, thus constituting further validation of the experiment for these four additional genes. The protective sucrose plus atrazine medium induced the expression of these transcription factors whereas the atrazine treatment had little effect. This increase in expression in the presence of atrazine plus sucrose started as soon as 4 h for At5g59820 (zinc finger protein Zat12) and At3g61630 (AP2 domain-containing TF). The AP2 domain-containing TF expression level remained relatively high all along the time course, while At5g59820 presented a constant increase of expression until 16 h. Expression level of At1g42990 (bZIP family protein AtbZIP60) and At3g56710 (Sigma factor binding protein SIB1) increased steadily with a peak of expression after 12 h of treatment in the presence of sucrose and atrazine. Conversely, the atrazine treatment repressed or did not modify gene expression relatively to the mannitol control, except at 16 h for At5g59820 and At3g61630 which presented a slight induction. These inductions during the first hours of sucrose plus atrazine treatment were consistent with an essential role of these TFs in the regulation of sugar-induced atrazine tolerance. It was also particularly interesting to identify early-induced genes such as At5g59820 and At3g61630.

## Discussion

Gene regulation was shown to be affected on a large scale by atrazine treatment, prior to the development of atrazine injury, in accordance with previous studies of exposure to other toxicants such as 2,4-D [65] or explosives [66]. Moreover, a number of these gene expression effects (Figure 2), such as repression of ROS defence mechanisms or repression of protein translation, were found to be potential components of atrazine sensitivity. This is, to our knowledge, the first report that atrazine can cause such large-scale primary and deleterious effects at the gene expression level.

**Figure 5:**
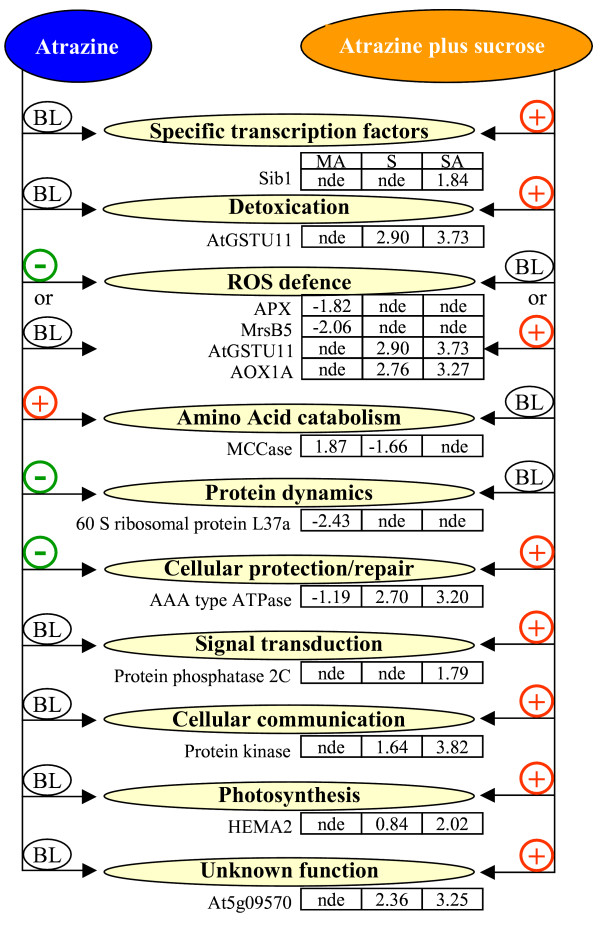
Major pathways implicated in sucrose-induced atrazine tolerance and in atrazine injury. Gene examples are given with their log_2_(ratio) for mannitol plus atrazine versus mannitol (MA), sucrose versus mannitol (S) and sucrose plus atrazine versus mannitol (SA) comparison. + indicates induction of expression, - indicates repression and BL indicates unchanged expression compared to the baseline level, nde: not differentially expressed; genes with a Bonferroni *P*-values higher than 0.05 were considered as being not differentially expressed as described in Lurin *et al*. [75].

In contrast, sucrose-induced atrazine-tolerance was characterized, on the one hand by the induction of several pathways linked to transcription, ROS defence, cellular repair and protection, signal transduction, cellular communication, photosynthesis and unknown functions, and on the other hand by a return to basal level of atrazine-affected pathways (Figure 5). Global analysis clearly showed that the transcriptome of Arabidopsis plantlets under conditions of sucrose-atrazine treatment was not the mere addition of atrazine-dependent and of sucrose-dependent transcriptomes. Thus, the number of genes specifically responding to sucrose-atrazine treatment amounted to nearly one third of all the genes that were differentially regulated under the sucrose plus atrazine treatment. The observed sucrose-atrazine transcriptome was therefore likely to depend on interacting signalling between sucrose and atrazine or between sucrose-derived and atrazine-derived, possibly ROS-related, stimuli. Soluble sugars have been involved in a number of stress-related processes, as metabolites that accumulate in response to stress conditions [67], as substrates of carbon metabolism [47], and as signals modifying gene expression [47,68]. Whereas, in some situations such as carbohydrate starvation, glucose and sucrose appear to have similar effects on gene expression [12], in some abiotic stress responses, such as response to anoxia [47], synthesis of anthocyanins [69], and response to PSII herbicides [7], sucrose treatment is more efficient than glucose treatment in providing protection mechanisms against the stress condition. These differential effects of glucose and sucrose greatly contrast with the similar effects of metabolisable sugars on the hexokinase-mediated signalling pathway [11,12], and would therefore agree with the existence of hexokinase-independent sucrose-specific signalling pathways, which have been postulated [70], but have remained elusive up to now, except for the characterization of a small number of transcription factors like the ATB2-type subgroup of AtbZIP TFs [71]. In our study, sucrose-specific TFs showed patterns of mRNA-level repression by sucrose alone, which were coherent with previous studies. Thus, the ATB2/AtbZIP factor, AtbZIP1 (At5g49450, group IV), was repressed by the sucrose treatment (Additional file 6). Such *ATB2 *transcripts are subjected to sucrose-induced repression of translation, resulting in a low level of ATB2 protein at 80 mM sucrose [71]. Target genes of these ATB2 TFs are expected to be specifically sucrose-responsive [71].

Interestingly, atrazine alone and the combined presence of sucrose and atrazine resulted in derepression of the sucrose-repressed AtbZIP1 transcription factor. The differences between the sucrose-atrazine and the individual sucrose transcriptomes were therefore strongly reflected at the level of TF gene expression. It thus seemed that development of tolerance response depended on sets of original TFs integrating signals from sucrose and from atrazine. The ability of atrazine to generate ROS [1] and the existence of ROS-signalling pathways [72] could suggest that atrazine-related signalling may involve ROS signalling. Moreover, our results (Table 4) strongly suggest interactions with salicylate, ABA and jasmonate signalling pathways. Since abiotic stress situations seem to involve signalling interactions between sugar and ethylene [43], it is therefore clear that the analysis of signalling pathways involved in sucrose-atrazine effects should use a panel of mutants affected not only in sugar signalling, but also in ethylene, salicylate, ABA and jasmonate signalling.

Since the initial ROS generated by atrazine is ^1^O_2 _[1], it may have been expected to observe similar gene expression modifications between atrazine treatment and the effects of the *flu *mutation, which results in higher ^1^O_2 _production upon dark/light shifts [5]. However, important differences were identified. For instance, the allene oxide synthase gene (At5g42650, group III) (Table 2), which is upregulated in the *flu *mutant, was strongly downregulated (7-fold repression) in the atrazine treatment. These differences could be ascribed to differences of ROS production and dynamics in terms of the chemical nature of generated ROS, in terms of time-course of accumulation, or in terms of signal intensity, between the atrazine treatment and the *flu *mutation. However, since the overall effect of the atrazine treatment seemed to result in partial induction of some ROS defence genes and in unexpected repression of other important ROS defence genes [5,31], such as the cytosolic APX1 (At1g07890, group III) and allene oxide synthase (At5g42650, group III), it was tempting to speculate that a xenobiotic signalling-pathway may exist. Even though xenobiotic-related signalling pathways are strongly suspected to exist in plants [65,66,73], none of them has yet been identified, in contrast with the detailed identification of xenobiotic receptors in animal cells. It would thus be interesting to investigate whether atrazine and other xenobiotics also develop their toxicity through signalling effects leading to the silencing or overcoming of ROS stress defences. Thus, the cytosolic *APX1 *(At1g07890, group III), which is a central component of the ROS gene network according to Davletova *et al*. [31], was the most significantly atrazine-repressed gene among the six genes of the APX family.

In contrast, under the situation of protection combining sucrose and atrazine, the most expressed gene was At2g21640 (group V), which corresponds to an expressed protein of unknown function, that has been found to be upregulated in most experiments of oxidative stress [24]. Moreover, TFs, which are strongly induced by atrazine plus sucrose, such as At3g50260 (group V, AP2 domain-containing factor), At2g30250 (group V, WRKY family), and At5g13080 (group V, WRKY family), have been described as common ROS-upregulated TFs [24]. These three TFs were also significantly, but to a lesser extent, upregulated by sucrose alone. In other words, sucrose appeared to be useful in re-establishing the expression of TFs that may be important for ROS defence. Sucrose lifting of atrazine-mediated TF repression was apparently not sufficient to establish tolerance to atrazine, since other TFs were significantly enhanced by the combination of sucrose and atrazine. Such is particularly the case for the At5g59820 (group V) and At1g42990 (group V) genes encoding respectively the zinc finger protein Zat12 and AtbZIP60 (Table 5). Both these TFs are likely to be important for atrazine tolerance, since Zat12 and AtbZIP60 have been respectively involved in the response to oxidative stress [60] and in the regulation of endoplasmic stress response [59]. Moreover, our results show that these genes were sequentially expressed in the course of the sucrose plus atrazine treatment (Figure 4), with Zat12 induction preceding that of AtbZIP60.

The set of TFs that is induced by the sucrose-atrazine treatment results in typical differential expression of multigene families that are involved in oxidative and xenobiotic stress responses, such as the GST multigene family. Besides the effects on ROS defence, sucrose treatment also allows plants to accumulate very high levels of atrazine in root and shoot tissues [9]. Whereas complete mineralization of atrazine does not seem to occur in sucrose-treated plants, radiolabelling experiments strongly suggest that conjugation processes are likely to occur [9]. According to the different mechanisms described in higher plants, these processes could involve conjugation to glutathione, glucose or macromolecular cell wall components [9]. Among the different pathways that may be involved in conjugation, the strongest differential effects of sucrose-atrazine treatment affected the P450 and GST multigene families, thus emphasising the importance of further work on the possibility of sucrose-induced atrazine-glutathione conjugation in Arabidopsis. These mechanisms, which depend on gene induction, would facilitate atrazine accumulation, while hampering binding of free atrazine to PSII, thus resulting in the observed maintenance of photosynthetic efficiency [7].

## Conclusion

The comparison of the atrazine-induced stress situation and of the sucrose-atrazine protection situation through the transcriptomic approach therefore sheds a new light on xenobiotic-signalling pathways, on xenobiotic tolerance pathways, and in identifying novel xenobiotic tolerance pathways, that would have otherwise, in the presence of lethal concentrations of atrazine, remained cryptic.

The study of early TFs and target genes involved in sucrose-induced tolerance will therefore be useful to investigate whether sugar-induced tolerance towards other xenobiotics involves the same general mechanism. Preliminary results indicate that sucrose can induce tolerance towards other xenobiotics.

## Methods

### Plant material and growth conditions

Seeds of *Arabidopsis thaliana *(ecotype Colombia, Col0) were surfaced-sterilized in bayrochlore/ethanol (1/1, v/v), rinsed in absolute ethanol and dried overnight. Germination and growth were carried out under axenic conditions. After seeds were sowed, they were placed at 4°C for 48 h in order to break dormancy and homogenize germination, and transferred at 22°C under a 16 h light period regime at 4500 lux until plantlets reached the 1.02 development stage [20]. Growth medium consisted of 0.8% agar in Murashige and Skoog basal salt mix (Sigma, St Louis, MO, USA) adjusted to pH 5.7. After cultivation, plantlets were transferred to fresh medium containing sucrose (80 mM) or mannitol (80 mM) in the absence or presence of 10 μM atrazine. Sucrose or mannitol were directly added during preparation of Murashige and Skoog agar media prior to sterilisation. Atrazine was sterilized by microfiltration through 0.2 μm cellulose acetate filters (Polylabo, Strasbourg, France) and then axenically added to melted Murashige and Skoog agar medium at a concentration of 10 μM.

### Measurement of seedling growth and development

Primary root length of plantlets was measured on vertical plates. Pigments were extracted by pounding aerial parts of plantlets in 80% acetone, and absorbance of the resulting extracts was measured at 663 nm, 646 nm and 470 nm. Chlorophyll and total carotenoid (xanthophylls and carotenes) levels in these extracts were determined from the equations given by Lichtenthaler and Wellburn [74].

### Measurement of photosynthesis parameters

Chlorophyll fluorescence and maximum PSII efficiency (F_v_/F_m_) were measured with a PAM-210 chlorophyll fluorometer system (Heinz Walz, Effeltrich, Germany). After dark adaptation during 30 min, minimum fluorescence (F_0_) was determined under weak red light. Maximum fluorescence of dark-adapted leaf (F_m_) was measured under a subsequent saturating pulse of red light, and variable fluorescence (F_v _= F_m _- F_0_) was determined [7].

### RNA isolation and microarray analysis

For the transcriptome studies, microarray analysis was performed with the CATMA array [17], which is especially dedicated to *Arabidopsis thaliana *and contains 24576 gene-specific tags corresponding to 22 089 genes plus 516 chloroplastic and mitochondrial probes [17]. The GST amplicons were purified on Multiscreen plates (Millipore, Bedford, MA, USA) and resuspended in TE-DMSO at 100 ng μl^-1^. The purified probes were transferred to 1536-well plates with a Genesis workstation (TECAN, Männedorf, Switzerland) and spotted on UltraGAPS slides (Corning, New York, NY, USA) using a Microgrid II (Genomic Solution, Huntingdon, UK).

The transcriptome analysis compared RNA of plantlets transferred on treatment medium during 24 h. Treatment media were: 80 mM mannitol (M, osmotic control and reference), 80 mM mannitol and 10 μM atrazine (MA, herbicide treatment), 80 mM sucrose (S, control medium) and 80 mM sucrose and 10 μM atrazine (SA, tolerance medium). The four conditions (M, S, MA, SA) were compared pairwise, so that the complete analysis consisted of 6 comparisons (Additional file 5).

Each treatment was repeated three times in independent experiments. In each experiment, 60 plantlets corresponding to a given treatment were harvested, frozen in liquid nitrogen and extracted for RNA. Total RNA was extracted using TRI Reagent^® ^(Sigma, St. Louis, MO, USA) following the manufacturer's protocol. Quantification, high quality and integrity of each RNA sample were verified by spectrophotometry and with the Agilent Bioanalyser (Waldbroon, Germany). For each treatment, RNAs from three independent biological experiments were pooled. cRNAs were produced from 2 μg of total RNA from each pool with the 'MessageAmp™ aRNA' kit (Ambion, Austin, TX, USA). Then, 5 μg of cRNAs was reverse transcribed in the presence of 200 U of SuperScript™ II (Invitrogen, Carlsbad, CA, USA), cy3-dUTP and cy5-dUTP (NEN, Boston, MA, USA). Samples were combined, purified and concentrated with YM30 Microcon columns (Millipore). For each of the six comparisons, two technical replicates with fluorochrome reversal were performed for each pool of RNA (i.e one dye swap per comparison). Each pool of three RNA samples corresponding to a given treatment was therefore hybridised six times. Slides were pre-hybridised for 1 h and hybridised overnight at 42°C in 25% formamide, as described by Lurin *et al.*[75]. The arrays were scanned on a GenePix 4000 A scanner (Axon Instruments, Foster City, CA, USA) and images were analysed by GenePix Pro 3.0 (Axon Instruments).

### Statistical analysis of microarray data

Statistical analysis, which was carried out by the statistics group of the Unité de Recherche en Génomique Végétale (URGV, Evry), followed the same design as described in several previous publications, such as Lurin *et al.*[75]. The statistical analysis was based on one dye-swap per comparison. For each array, the raw data comprised the logarithm of median feature pixel intensity at wavelengths 635 nm (red) and 532 nm (green). No background was subtracted. In the following description, log ratio refers to the differential expression between the different treatments. It is either log_2_(red/green) or log_2_(green/red) according to the experimental design. An array-by-array normalisation was performed to remove systematic biases. First, we excluded spots that were considered to show badly formed features. Then, we performed a global intensity-dependent normalisation using the loess procedure [76] to correct the dye bias. Finally, on each block, the log-ratio median was subtracted from each value of the log-ratio of the block to correct any print-tip effect on each metablock. In order to determine differentially expressed genes, we performed a paired t-test on the log-ratios, assuming that the variance of the log-ratios is the same for all genes. Spots displaying extremes of specific variance (too small or too large) were excluded. The raw *P*-values were adjusted by the Bonferroni method, which controls the Family Wise Error Rate (FWER) [77]. We considered as being differentially expressed the genes with a Bonferroni *P*-value ≤ 0.05 as described in Lurin *et al*. [75]. The data were deposited in ArrayExpress [78] (E-MEXP-411) according to the MIAME standards proposed by the Microarray Gene Expression Data (MGED) Society [79].

### Microarray data validation and qRT-PCR experiment

qRT-PCR experiments were carried out with cDNA synthesised (Iscript™ cDNA Synthesis kit, Bio-Rad, Hercules, CA, USA) from the cRNA used in the microarray analysis and from total RNA isolated from independent experiments, where plantlets were transferred as indicated on mannitol (80 mM) (M), mannitol (80 mM) plus atrazine (10 μM) (MA) and sucrose (80 mM) plus atrazine (10 μM) (SA). Resulting cDNAs were used to determine expression profiles according to the different treatments. Quantitative PCR was performed using iQ™ SYBR Green Supermix (Bio-Rad, Hercules, CA, USA). Conditions were as follows: 95°C 3 min, and 40 (95°C 15 sec, 60°C 45 sec) cycles. All reactions were performed in triplicate. Specific primers for each gene selected for analysis were designed using Beacon Designer 5.0 software (Additional file 12). The results of the analysis were treated with Gene Expression version 1.1 software. For real-time RT-PCR validation, the log_2_(relative expression to the control sample M) of the qRT-PCR was compared with the log_2_(intensity ratio) of the array analysis. The real-time RT-PCR time-course experiment takes as references appropriate controls (Mannitol-treated plants harvested at the same time point).

### Analysis of microarray data

Functional categories of differentially expressed genes were adapted from the categories defined by the Munich Information Center for Protein Sequences (MIPS) [21]. For analysis of gene-expression data across the different experiments, hierarchical clustering was performed with the Genesis software [80] using Euclidian distance for the similarity distance and the average linkage clustering for the linkage rule. The AtcisDB database [63] was used for analysis of cis-acting regulatory elements in gene promoters.

## Abbreviations used

ABA, abscisic acid; PSII, photosystem II; ROS, reactive oxygen species; qRT-PCR, quantitative real-time reverse transcription-polymerase chain reaction; TF, transcription factor; GST, glutathione S-transferase.

## Authors' contributions

FR, IC and GG conceived the study and designed experiments, CS, FCH, AEA and JPR contributed in the conception of the study, FR, CS and GG performed the experiments, JPR coordinated the microarray analysis at the Unité de Recherche en Génomique Végétale (URGV) using the Complete Arabidopsis Transcriptome MicroArray (CATMA), LT performed microarray experiments, MLMM carried out statistical analysis for microarray data, FR, IC and GG carried out analysis and interpretation of experimental data including bioinformatics analyses. The manuscript was written by FR, IC, GG and read and revised by all other authors.

## Supplementary Material

Additional file 1 Physiological effects of atrazine and sucrose treatments. Arabidopsis plantlets were grown on Murashige and Skoog agar medium and transferred at the 1.02 development stage [20] to Murashige and Skoog agar medium supplemented with mannitol (80 mM), mannitol (80 mM) plus atrazine (10 μM), sucrose (80 mM) and sucrose (80 mM) plus atrazine (10 μM). Pictures were taken during 8 days after transfer. Click here for file

Additional file 2 Effects of atrazine and sucrose treatments on chlorophyll content. Arabidopsis plantlets transferred to Murashige and Skoog agar medium supplemented with mannitol (80 mM)(A), sucrose (80 mM)(B), mannitol (80 mM) plus atrazine (10 μM)(C), and sucrose (80 mM) plus atrazine (10 μM)(D) were harvested after 0, 1, 4 and 8 days of treatment for pigment determination. Values are the mean (± S.E.M.) of measurements on at least 10 plantlets. Click here for file

Additional file 3 Effects of atrazine and sucrose treatments on carotenoid content. Arabidopsis plantlets transferred to Murashige and Skoog agar medium supplemented with mannitol (80 mM)(A), sucrose (80 mM)(B), mannitol (80 mM) plus atrazine (10 μM)(C), and sucrose (80 mM) plus atrazine (10 μM)(D) were harvested after 0, 1, 4 and 8 days of treatment for pigment determination. Values are the mean (± S.E.M.) of measurements on at least 10 plantlets. Click here for file

Additional file 4 Effects of atrazine and sucrose treatments on photosystem II efficiency (Fv/Fm). Arabidopsis plantlets were transferred to Murashige and Skoog agar medium supplemented with mannitol (80 mM)(A), sucrose (80 mM)(B), mannitol (80 mM) plus atrazine (10 μM)(C), and sucrose (80 mM) plus atrazine (10 μM)(D); chlorophyll fluorescence and maximum PSII efficiency (F_v_/F_m_) were measured after 0, 1, 4 and 8 days of treatment. Values are the mean (± S.E.M.) of measurements on at least 10 plantlets. Click here for file

Additional file 5 Schematic representation of experimental procedure for end-point CATMA array analysis. Six comparisons of mannitol-, sucrose-, mannitol-atrazine- and sucrose-atrazine-treated plantlets were performed at the end of a 24 h treatment. Double arrows indicate dye-swap hybridisation. Click here for file

Additional file 6 Overview of the 810 highly-responsive genes. Additional file 6 lists genes whose log_2_(ratio) was greater than 1.585 or lesser than -1.585 (corresponding to 3-fold change) in at least one of the MA/M, SA/M or S/M comparisons in the CATMA array experiment. The full data were deposited in ArrayExpress [78] (E-MEXP-411). Click here for file

Additional file 7 Validation of microarray results using real-time PCR. The log_2_(relative quantity to the control sample mannitol) of the qRT-PCR (open) obtained for 8 selected genes was compared with the log_2_(intensity ratio) of the CATMA array analysis (striped) using RNA from plantlets transferred during 24 h to mannitol-atrazine (blue), sucrose (red) or sucrose-atrazine (orange) treatments. Selected genes were: 4-hydroxyphenylpyruvate dioxygenase (PDS1) (At1g06570), Glutathione dehydrogenase (ascorbate) (At1g75270), Carbohydrate transporter/sugar porter (At1g77210), CYP710A1 (At2g34500), ATPase (At2g47000), Glutathione transferase (At3g09270), Glucose-6-phosphate dehydrogenase (G6PD6) (At5g40760) and Chaperone GrpE-like protein (At5g55200). The Ubiquitin 5 (UB5) gene was taken as internal standard, and qRT-PCR was performed as described in Methods. Values are given as means (± SEM) of three technical replicates. This validation was repeated three times with independent biological samples, and gave similar trends. Click here for file

Additional file 8 Statistical significance of gene repression and induction for the different functional categories. The statistical analysis using a χ^2 ^test was realized on each functional category of genes in order to compare the significance of induction and repression between the different comparisons (MA/M, S/M, SA/M). Click here for file

Additional file 9 Repression by atrazine of genes in DNA and protein dynamics. Additional file 9 lists several genes involved in DNA and protein dynamics and repressed by atrazine treatment in the present study. Click here for file

Additional file 10 Sucrose treatment in *Arabidopsis thaliana*: comparison of its transcriptomic effects with previous studies. Additional file 10 lists several genes corresponding to typical markers of carbohydrate responses according to previous studies and found responsive to sucrose treatment in the present study. Click here for file

Additional file 11 Conserved cis-acting elements and their percentage of occurrence in the promoter region of genes presenting high induction by sucrose-atrazine combination. Additional file 11 lists cis-acting regulatory elements and their occurrence in promoters of genes presenting high induction by the sucrose-atrazine combination and corresponding transcription factors. Analysis of cis-acting regulatory elements was carried out with the AtcisDB database Click here for file

Additional file 12 Genes selected for qRT-PCR analysis and primer sequences. Additional file 12 lists genes selected for qRT-PCR analysis and primer sequences. Click here for file

## References

[B1] Rutherford AW, Krieger-Liszkay A (2001). Herbicide-induced oxidative stress in photosystem II. Trends Biochem Sci.

[B2] Nishiyama Y, Allakhverdiev SI, Yamamoto H, Hayashi H, Murata N (2004). Singlet oxygen inhibits the repair of photosystem II by suppressing the translation elongation of the D1 protein in Synechocystis sp. PCC 6803. Biochemistry.

[B3] Rinalducci S, Pedersen JZ, Zolla L (2004). Formation of radicals from singlet oxygen produced during photoinhibition of isolated light-harvesting proteins of photosystem II. Biochim Biophys Acta.

[B4] Ryter SW, Tyrrell RM (1998). Singlet molecular oxygen (^1^O_2_): a possible effector of eukaryotic gene expression. Free Radical Bio Med.

[B5] op den Camp RG, Przybyla D, Ochsenbein C, Laloi C, Kim C, Danon A, Wagner D, Hideg E, Gobel C, Feussner I, Nater M, Apel K (2003). Rapid induction of distinct stress responses after the release of singlet oxygen in *Arabidopsis*. Plant Cell.

[B6] Nemat Alla MM, Hassan NM (2006). Changes of antioxidants levels in two maize lines following atrazine treatments. Plant Physiol Biochem.

[B7] Sulmon C, Gouesbet G, Couée I, El Amrani A (2004). Sugar-induced tolerance to atrazine in Arabidopsis seedlings: interacting effects of atrazine and soluble sugars on *psbA *mRNA and D1 protein levels. Plant Science.

[B8] Sulmon C, Gouesbet G, El Amrani A, Couée I (2006). Sugar-induced tolerance to the herbicide atrazine in Arabidopsis seedlings involves activation of oxidative and xenobiotic stress responses. Plant Cell Rep.

[B9] Sulmon C, Gouesbet G, Binet F, Martin-Laurent F, El Amrani A, Couée I (2007). Sucrose amendment enhances phytoaccumulation of the herbicide atrazine in *Arabidopsis thaliana*. Environ Pollut.

[B10] Koch KE (1996). Carbohydrate-modulated gene expression in plants. Annu Rev Plant Physiol Plant Mol Biol.

[B11] Rolland F, Baena-Gonzalez E, Sheen J (2006). Sugar sensing and signaling in plants: conserved and novel mechanisms. Annu Rev Plant Biol.

[B12] Pego JV, Kortstee AJ, Huijser C, Smeekens SC (2000). Photosynthesis, sugars and the regulation of gene expression. J Exp Bot.

[B13] Li H, Sherman LA (2000). A redox-responsive regulator of photosynthesis gene expression in the cyanobacterium *Synechocystis *sp. strain PCC 6803. J Bacteriol.

[B14] Alfonso M, Perewoska I, Kirilovsky D (2000). Redox control of *psbA *gene expression in the cyanobacterium Synechocystis PCC 6803. Involvement of the cytochrome b(6)/f complex. Plant Physiol.

[B15] Pfannschmidt T, Schutze K, Brost M, Oelmuller R (2001). A novel mechanism of nuclear photosynthesis gene regulation by redox signals from the chloroplast during photosystem stoichiometry adjustment. J Biol Chem.

[B16] Thum KE, Shin MJ, Palenchar PM, Kouranov A, Coruzzi GM (2004). Genome-wide investigation of light and carbon signaling interactions in Arabidopsis. Genome Biol.

[B17] Hilson P, Allemeersch J, Altmann T, Aubourg S, Avon A, Beynon J, Bhalerao RP, Bitton F (2004). Versatile gene-specific sequence tags for Arabidopsis functional genomics: transcript profiling and reverse genetics applications. Genome Res.

[B18] Sulmon C, Gouesbet G, Couée I, El Amrani A (2005). Method for improving the phytoremediation of polluted sites by providing plants with exogenous carbohydrates. Office Européen des Brevets (OEB), International Patent, WO2005025769 A1.

[B19] Sulmon C, Gouesbet G, Couée I, El Amrani A (2007). Method for improving the phytoremediation of polluted sites by providing plants with exogenous carbohydrates. United States Patent and Trademark Office, US Patent, US 2007/0093388 A1.

[B20] Boyes DC, Zayed AM, Ascenzi R, McCaskill AJ, Hoffman NE, Davis KR, Gorlach J (2001). Growth stage-based phenotypic analysis of Arabidopsis: a model for high throughput functional genomics in plants. Plant Cell.

[B21] Munich Information Centre for Protein Sequence database (MIPS). http://mips.gsf.de/projects/funcat.

[B22] Ruepp A, Zollner A, Maier D, Albermann K, Hani J, Mokrejs M, Tetko I, Guldener U, Mannhaupt G, Munsterkotter M, Mewes HW (2004). The FunCat, a functional annotation scheme for systematic classification of proteins from whole genomes. Nucleic Acids Res.

[B23] Gonzali S, Loreti E, Solfanelli C, Novi G, Alpi A, Perata P (2006). Identification of sugar-modulated genes and evidence for *in vivo *sugar sensing in Arabidopsis. J Plant Res.

[B24] Gadjev I, Vanderauwera S, Gechev TS, Laloi C, Minkov IN, Shulaev V, Apel K, Inze D, Mittler R, Van Breusegem F (2006). Transcriptomic footprints disclose specificity of reactive oxygen species signaling in Arabidopsis. Plant Physiol.

[B25] Wang PC, Du YY, An GY, Zhou Y, Miao C, Song CP (2006). Analysis of global expression profiles of Arabidopsis genes under abscisic acid H_2_O_2 _applications. J Integr Plant Biol.

[B26] Scandalios JG (2002). Oxidative stress responses – what have genome-scale studies taught us?. Genome Biol.

[B27] Diener AC, Gaxiola RA, Fink GR (2001). Arabidopsis ALF5, a multidrug efflux transporter gene family member, confers resistance to toxins. Plant Cell.

[B28] Dixon DP, Cummins I, Cole DJ, Edwards R (1998). Glutathione-mediated detoxification systems in plants. Curr Opin Plant Biol.

[B29] Norris SR, Barrette TR, DellaPenna D (1995). Genetic dissection of carotenoid synthesis in Arabidopsis defines plastoquinone as an essential component of phytoene desaturation. Plant Cell.

[B30] Telfer A, Dhami S, Bishop SM, Phillips D, Barber J (1994). β-Carotene quenches singlet oxygen formed by isolated photosystem II reaction centers. Biochemistry.

[B31] Davletova S, Rizhsky L, Liang H, Shengqiang Z, Oliver DJ, Coutu J, Shulaev V, Schlauch K, Mittler R (2005). Cytosolic ascorbate peroxidase 1 is a central component of the reactive oxygen network of Arabidopsis. Plant Cell.

[B32] Wagner U, Edwards R, Dixon DP, Mauch F (2002). Probing the diversity of the Arabidopsis glutathione S-transferase gene family. Plant Mol Biol.

[B33] Romero HM, Berlett BS, Jensen PJ, Pell EJ, Tien M (2004). Investigations into the role of the plastidial peptide methionine sulfoxide reductase in response to oxidative stress in Arabidopsis. Plant Physiol.

[B34] Vieira Dos Santos C, Cuine S, Rouhier N, Rey P (2005). The Arabidopsis plastidic methionine sulfoxide reductase B proteins. Sequence and activity characteristics, comparison of the expression with plastidic methionine sulfoxide reductase A, and induction by photooxidative stress. Plant Physiol.

[B35] Vignols F, Brehelin C, Surdin-Kerjan Y, Thomas D, Meyer Y (2005). A yeast two-hybrid knockout strain to explore thioredoxin-interacting proteins in vivo. Proc Natl Acad Sci USA.

[B36] Meyer Y, Reichheld JP, Vignols F (2005). Thioredoxins in Arabidopsis and other plants. Photosynth Res.

[B37] Porankiewicz J, Wang J, Clarke AK (1999). New insights into the ATP-dependent Clp protease: *Escherichia coli *and beyond. Mol Microbiol.

[B38] Nakabayashi K, Ito M, Kiyosue T, Shinozaki K, Watanabe A (1999). Identification of *clp *genes expressed in senescing Arabidopsis leaves. Plant Cell Physiol.

[B39] Bernhard WR, Matile P (1994). Differential expression of glutamine synthetase genes during the senescence of *Arabidopsis thaliana *rosette leaves. Plant Sci.

[B40] Che P, Wurtele ES, Nikolau BJ (2002). Metabolic and environmental regulation of 3-methylcrotonyl-coenzyme A carboxylase expression in Arabidopsis. Plant Physiol.

[B41] Däschner K, Couée I, Binder S (2001). The mitochondrial isovaleryl-coenzyme a dehydrogenase of Arabidopsis oxidizes intermediates of leucine and valine catabolism. Plant Physiol.

[B42] Contento AL, Kim SJ, Bassham DC (2004). Transcriptome profiling of the response of Arabidopsis suspension culture cells to Suc starvation. Plant Physiol.

[B43] Price J, Laxmi A, Martin SKS, Jang JC (2004). Global transcription profiling reveals multiple sugar signal transduction mechanisms in Arabidopsis. Plant Cell.

[B44] Suzuki M, Ketterling MG, Li QB, McCarty DR (2003). Viviparous1 alters global gene expression patterns through regulation of abscisic acid signaling. Plant Physiol.

[B45] Lacomme C, Roby D (1996). Molecular cloning of a sulfotransferase in *Arabidopsis thaliana *and regulation during development and in response to infection with pathogenic bacteria. Plant Mol Biol.

[B46] Colangelo EP, Guerinot ML (2004). The essential basic helix-loop-helix protein FIT1 is required for the iron deficiency response. Plant Cell.

[B47] Loreti E, Poggi A, Novi G, Alpi A, Perata P (2005). A genome-wide analysis of the effects of sucrose on gene expression in Arabidopsis seedlings under anoxia. Plant Physiol.

[B48] Kürsteiner O, Dupuis I, Kuhlemeier C (2003). The *pyruvate decarboxylase1 *gene of Arabidopsis is required during anoxia but not other environmental stresses. Plant Physiol.

[B49] Umbach AL, Ng VS, Siedow JN (2006). Regulation of plant alternative oxidase activity: a tale of two cysteines. Biochim Biophys.

[B50] Morikawa T, Mizutani M, Aoki N, Watanabe B, Saga H, Saito S, Oikawa A, Suzuki H, Sakurai N, Shibata D, Wadano A, Sakata K, Ohta D (2006). Cytochrome P450 CYP710A encodes the sterol C-22 desaturase in Arabidopsis and Tomato. Plant Cell.

[B51] Vale RD (2000). AAA proteins. Lords of the ring. J Cell Biol.

[B52] Chen IP, Haehnel U, Altschmied L, Schubert I, Puchta H (2003). The transcriptional response of Arabidopsis to genotoxic stress – a high-density colony array study (HDCA). Plant J.

[B53] Barth C, Moeder W, Klessig DF, Conklin PL (2004). The timing of senescence and response to pathogens is altered in the ascorbate-deficient Arabidopsis mutant vitamin c-1. Plant Physiol.

[B54] Chen W, Provart NJ, Glazebrook J, Katagiri F, Chang HS, Eulgem T, Mauch F, Luan S, Zou G, Whitham SA, Budworth PR, Tao Y, Xie Z, Chen X, Lam S, Kreps JA, Harper JF, Si-Ammour A, Mauch-Mani B, Heinlein M, Kobayashi K, Hohn T, Dangl JL, Wang X, Zhu T (2002). Expression profile matrix of Arabidopsis transcription factor genes suggests their putative functions in response to environmental stresses. Plant Cell.

[B55] Savenstrand H, Brosche M, Strid A (2002). Regulation of gene expression by low levels of ultraviolet-B radiation in *Pisum sativum*: isolation of novel genes by suppression subtractive hybridisation. Plant Cell Physiol.

[B56] Hampton CR, Bowen HC, Broadley MR, Hammond JP, Mead A, Payne KA, Pritchard J, White PJ (2004). Cesium toxicity in Arabidopsis. Plant Physiol.

[B57] Riechmann JL, Heard J, Martin G, Reuber L, Jiang C, Keddie J, Adam L, Pineda O, Ratcliffe OJ, Samaha RR, Creelman R, Pilgrim M, Broun P, Zhang JZ, Ghandehari D, Sherman BK, Yu G (2000). Arabidopsis transcription factors: genome-wide comparative analysis among eukaryotes. Science.

[B58] Morikawa K, Shiina T, Murakami S, Toyoshima Y (2002). Novel nuclear-encoded proteins interacting with a plastid sigma factor, Sig1, in *Arabidopsis thaliana*. FEBS Lett.

[B59] Iwata Y, Koizumi N (2005). An Arabidopsis transcription factor, AtbZIP60, regulates the endoplasmic reticulum stress response in a manner unique to plants. Proc Natl Acad Sci USA.

[B60] Davletova S, Schlauch K, Coutu J, Mittler R (2005). The zinc-finger protein Zat12 plays a central role in reactive oxygen and abiotic stress signaling in Arabidopsis. Plant Physiol.

[B61] Nakashima K, Shinwari ZK, Sakuma Y, Seki M, Miura S, Shinozaki K, Yamaguchi-Shinozaki K (2000). Organization and expression of two *Arabidopsis DREB2 *genes encoding DRE-binding proteins involved in dehydration- and high-salinity-responsive gene expression. Plant Mol Biol.

[B62] Eulgem T, Rushton PJ, Robatzek S, Somssich IE (2000). The WRKY superfamily of plant transcription factors. Trends Plant Sci.

[B63] The Arabidopsis *cis*-regulatory element database AtcisDB). http://arabidopsis.med.ohio-state.edu/AtcisDB/.

[B64] Toufighi K, Brady SM, Austin R, Ly E, Provart NJ (2005). The Botany Array Resource: e-Northerns, Expression Angling, and promoter analyses. Plant J.

[B65] Teixeira MC, Duque P, Sa-Correia I (2007). Environmental genomics: mechanistic insights into toxicity of and resistance to the herbicide 2,4-D. Trends Biotechnol.

[B66] Ekman DR, Wolfe NL, Dean JF (2005). Gene expression changes in *Arabidopsis thaliana *seedling roots exposed to the munition hexahydro-1,3,5-trinitro-1,3,5-triazine. Environ Sci Technol.

[B67] Roitsch T (1999). Source-sink regulation by sugar and stress. Curr Opin Plant Biol.

[B68] Couée I, Sulmon C, Gouesbet G, El Amrani A (2006). Involvement of soluble sugars in reactive oxygen species balance and responses to oxidative stress in plants. J Exp Bot.

[B69] Teng S, Keurentjes J, Bentsink L, Koornneff M, Smeekens S (2005). Sucrose-specific induction of anthocyanin biosynthesis in Arabidopsis requires the MYB75/PAP1 gene. Plant Physiol.

[B70] Lalonde S, Boles E, Hellmann H, Barker L, Patrick JW, Frommer WB, Ward JM (1999). The dual function of sugar carriers. Transport and sugar sensing. Plant Cell.

[B71] Wiese A, Elzinga N, Wobbes B, Smeekens S (2005). Sucrose-induced translational repression of plant bZIP-type transcription factors. Biochem Soc T.

[B72] Mittler R, Vanderauwera S, Gollery M, Van Breusegem F (2004). Reactive oxygen gene network of plants. Trends Plant Sci.

[B73] Baerson SR, Sanchez-Moreiras A, Pedrol-Bonjoch N, Schulz M, Kagan IA, Agarwal AK, Reigosa MJ, Duke SO (2005). Detoxification and transcriptome response in Arabidopsis seedlings exposed to the allelochemical benzoxazolin-2(3H)-one. J Biol Chem.

[B74] Lichtenthaler HK, Wellburn AR (1983). Determinations of total carotenoids and chlorophylls *a *and *b *of leaf extracts in different solvents. Biochem Soc T.

[B75] Lurin C, Andres C, Aubourg S, Bellaoui M, Bitton F, Bruyere C, Caboche M, Debast C, Gualberto J, Hoffmann B, Lecharny A, Le Ret M, Martin-Magniette ML, Mireau H, Peeters N, Renou JP, Szurek B, Taconnat L, Small I (2004). Genome-wide analysis of Arabidopsis pentatricopeptide repeat proteins reveals their essential role in organelle biogenesis. Plant Cell.

[B76] Yang YH, Dudoit S, Luu P, Lin DM, Peng V, Ngai J, Speed TP (2002). Normalization for cDNA microarray data: a robust composite method addressing single and multiple slide systematic variation. Nucleic Acids Res.

[B77] Ge Y, Duboit S, Speed TP (2003). Resampling-based multiple testing for microarray data analysis. TEST.

[B78] ArrayExpress database. http://www.ebi.ac.uk/arrayexpress/.

[B79] The Microarray Gene Expression Data (MGED) Society. http://www.mged.org/.

[B80] Genesis software. http://genome.tugraz.at.

