# Supplementary figures and images for: Genome-wide interacting effects of sucrose and herbicide-mediated stress in Arabidopsis thaliana: novel insights into atrazine toxicity and sucrose-induced tolerance
Source: BMC Genomics. 2007 Dec 5;8:450. doi: 10.1186/1471-2164-8-450 (PMC2242805; doi:10.1186/1471-2164-8-450)

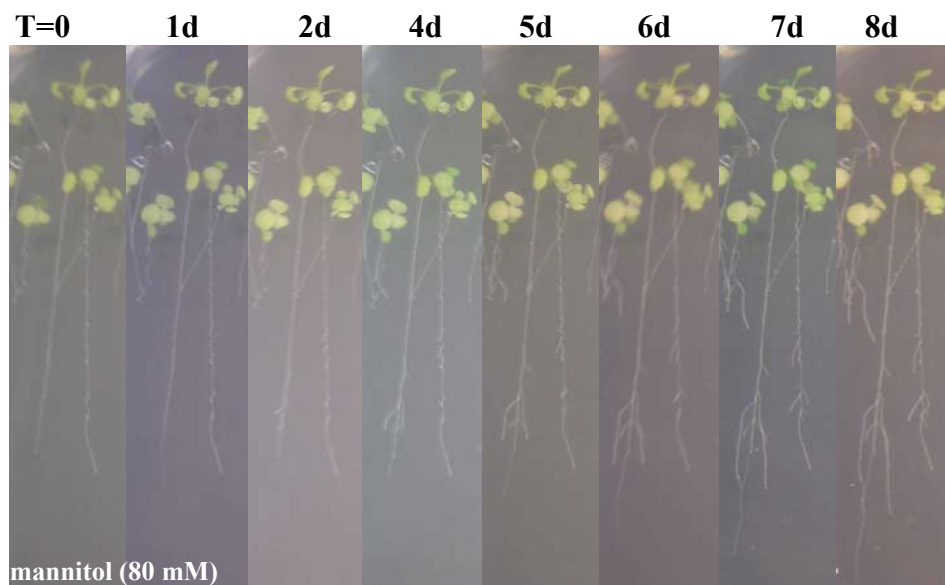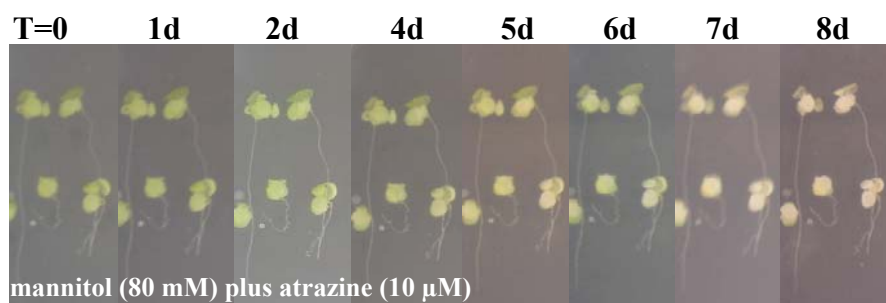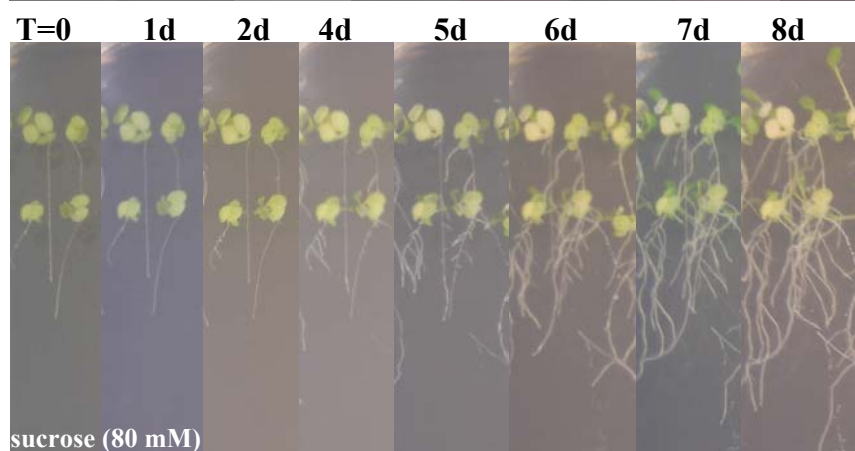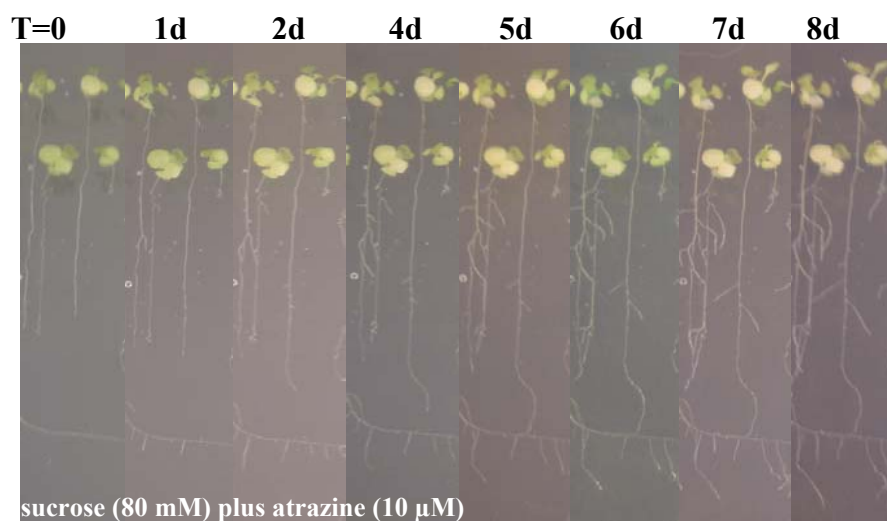

Supplement: Additional file 1 — Physiological effects of atrazine and sucrose treatments. Arabidopsis plantlets were grown on Murashige and Skoog agar medium and transferred at the 1.02 development stage [20] to Murashige and Skoog agar medium supplemented with mannitol (80 mM), mannitol (80 mM) plus atrazine (10 μM), sucrose (80 mM) and sucrose (80 mM) plus atrazine (10 μM). Pictures were taken during 8 days after transfer. [file 1471-2164-8-450-S1.pdf]

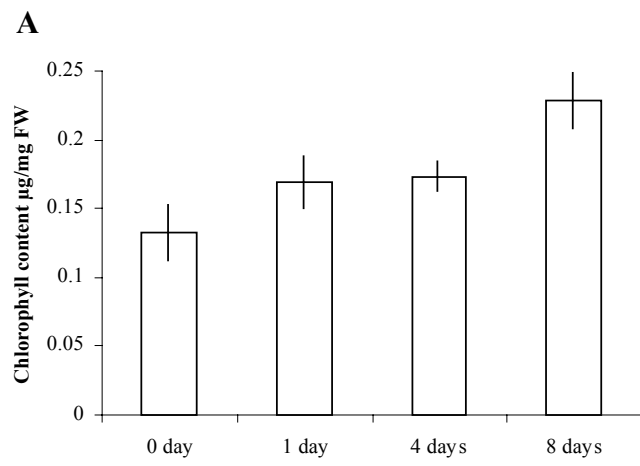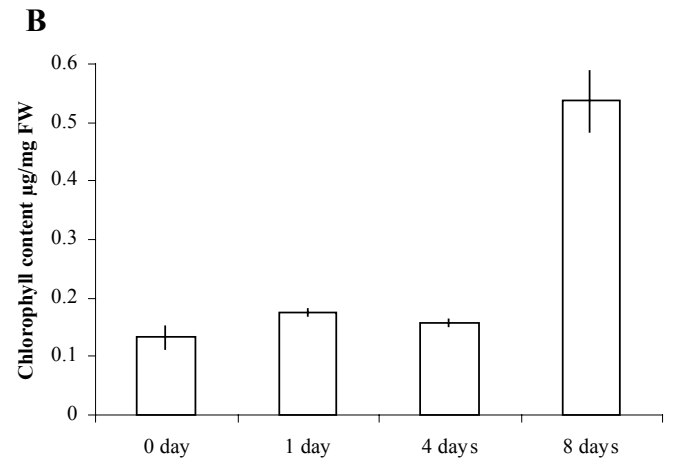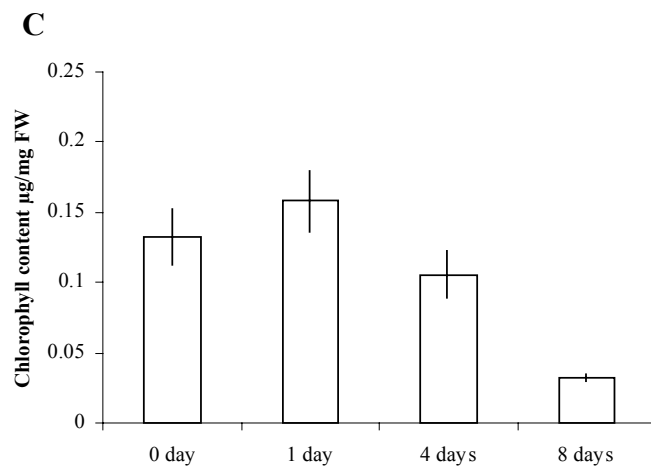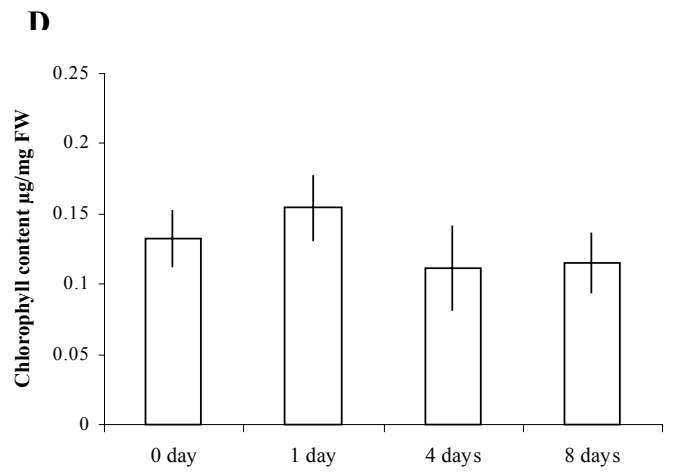

Supplement: Additional file 2 — Effects of atrazine and sucrose treatments on chlorophyll content. Arabidopsis plantlets transferred to Murashige and Skoog agar medium supplemented with mannitol (80 mM)(A), sucrose (80 mM)(B), mannitol (80 mM) plus atrazine (10 μM)(C), and sucrose (80 mM) plus atrazine (10 μM)(D) were harvested after 0, 1, 4 and 8 days of treatment for pigment determination. Values are the mean (± S.E.M.) of measurements on at least 10 plantlets. [file 1471-2164-8-450-S2.pdf]

**A**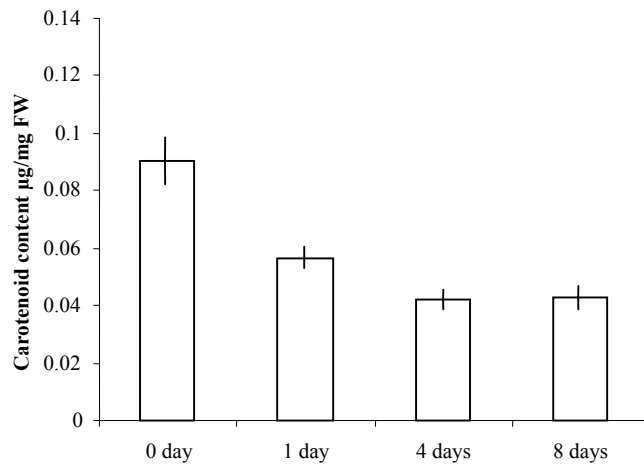**B**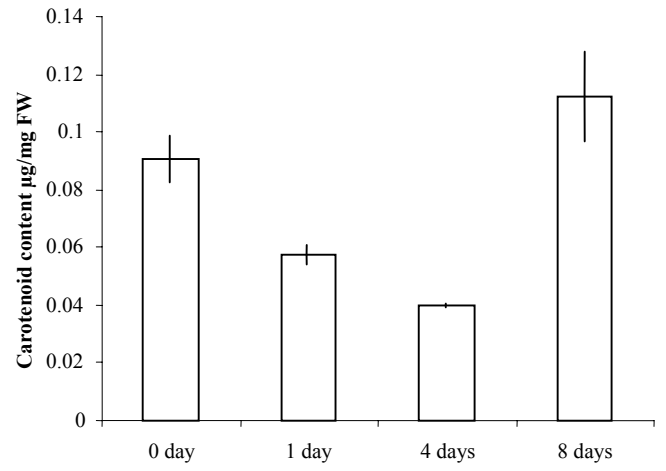**C**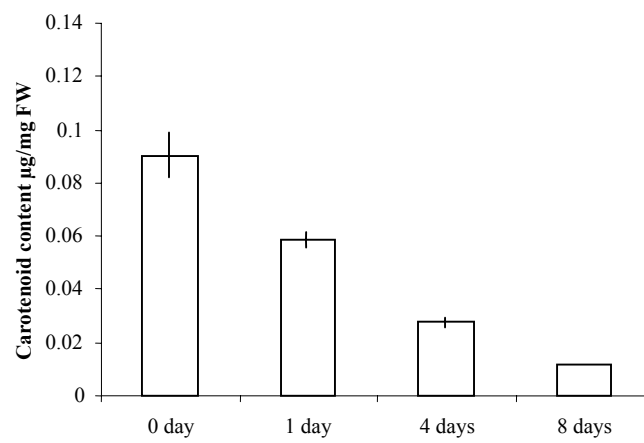**D**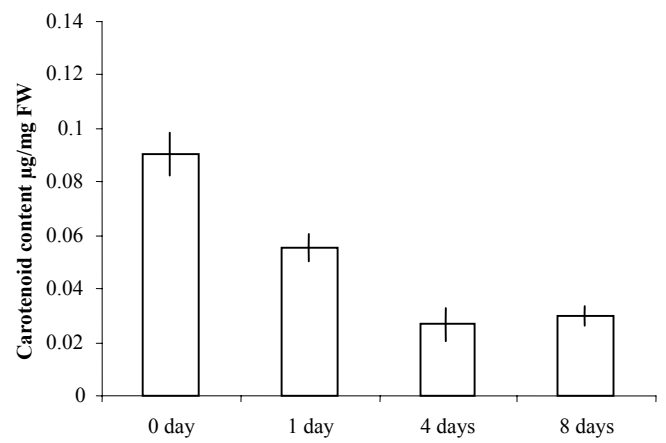

Supplement: Additional file 3 — Effects of atrazine and sucrose treatments on carotenoid content. Arabidopsis plantlets transferred to Murashige and Skoog agar medium supplemented with mannitol (80 mM)(A), sucrose (80 mM)(B), mannitol (80 mM) plus atrazine (10 μM)(C), and sucrose (80 mM) plus atrazine (10 μM)(D) were harvested after 0, 1, 4 and 8 days of treatment for pigment determination. Values are the mean (± S.E.M.) of measurements on at least 10 plantlets. [file 1471-2164-8-450-S3.pdf]

**A**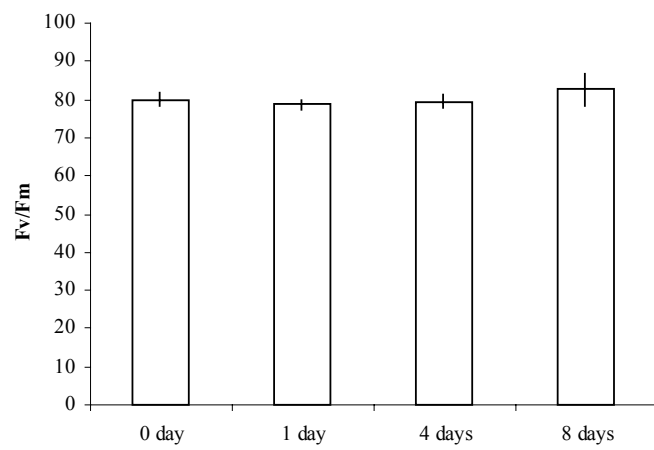**B**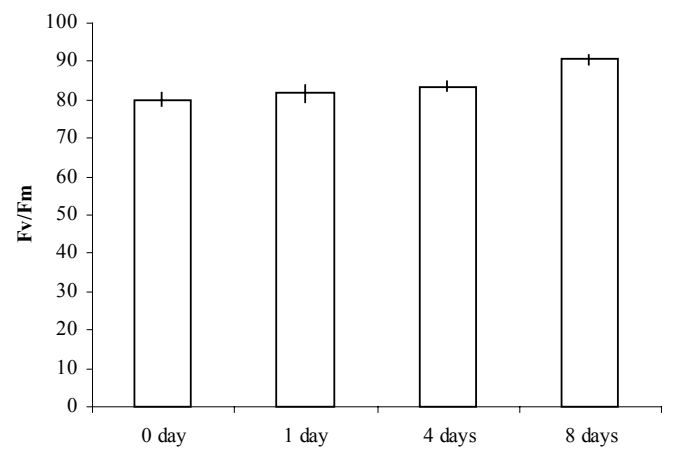**C**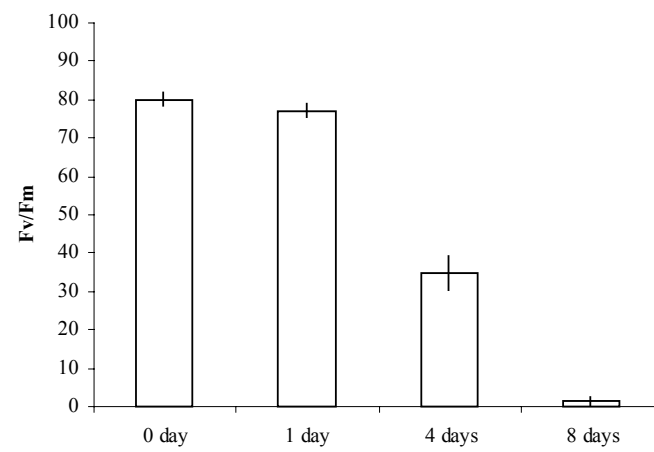**D**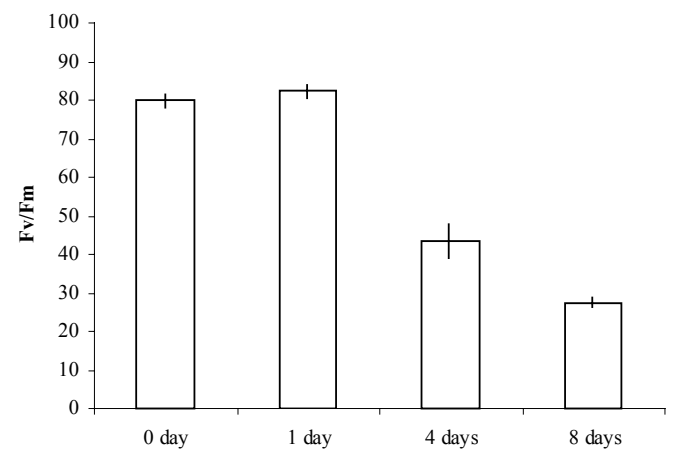

Supplement: Additional file 4 — Effects of atrazine and sucrose treatments on photosystem II efficiency (Fv/Fm). Arabidopsis plantlets were transferred to Murashige and Skoog agar medium supplemented with mannitol (80 mM)(A), sucrose (80 mM)(B), mannitol (80 mM) plus atrazine (10 μM)(C), and sucrose (80 mM) plus atrazine (10 μM)(D); chlorophyll fluorescence and maximum PSII efficiency (Fv/Fm) were measured after 0, 1, 4 and 8 days of treatment. Values are the mean (± S.E.M.) of measurements on at least 10 plantlets. [file 1471-2164-8-450-S4.pdf]

**Mannitol (M)       $\longleftrightarrow$       Sucrose (S)**

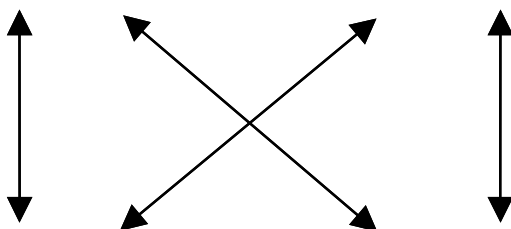

**Mannitol - Atrazine  
(MA)**

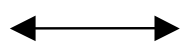

**Sucrose -Atrazine  
(SA)**

Supplement: Additional file 5 — Schematic representation of experimental procedure for end-point CATMA array analysis. Six comparisons of mannitol-, sucrose-, mannitol-atrazine- and sucrose-atrazine-treated plantlets were performed at the end of a 24 h treatment. Double arrows indicate dye-swap hybridisation. [file 1471-2164-8-450-S5.pdf]

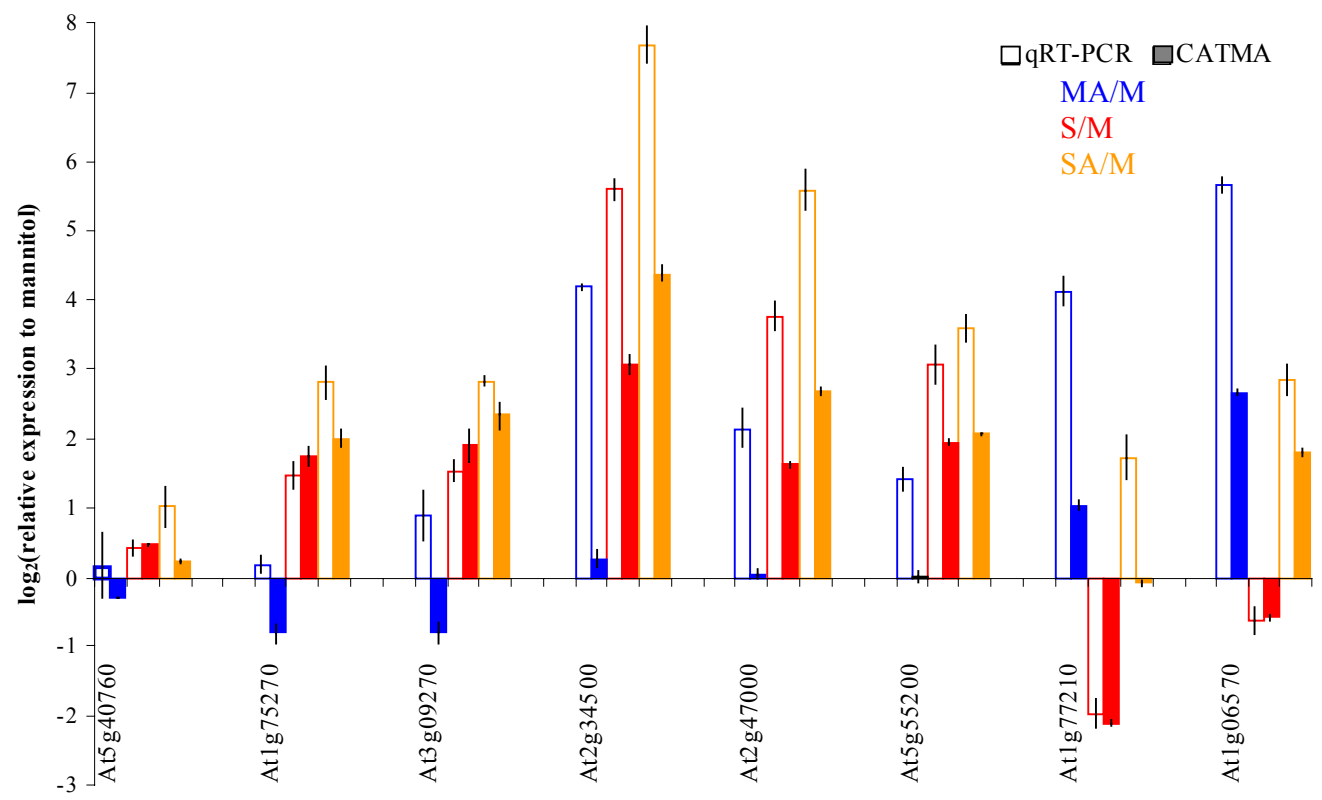

Supplement: Additional file 7 — Validation of microarray results using real-time PCR. The log2(relative quantity to the control sample mannitol) of the qRT-PCR (open) obtained for 8 selected genes was compared with the log2(intensity ratio) of the CATMA array analysis (striped) using RNA from plantlets transferred during 24 h to mannitol-atrazine (blue), sucrose (red) or sucrose-atrazine (orange) treatments. Selected genes were: 4-hydroxyphenylpyruvate dioxygenase (PDS1) (At1g06570), Glutathione dehydrogenase (ascorbate) (At1g75270), Carbohydrate transporter/sugar porter (At1g77210), CYP710A1 (At2g34500), ATPase (At2g47000), Glutathione transferase (At3g09270), Glucose-6-phosphate dehydrogenase (G6PD6) (At5g40760) and Chaperone GrpE-like protein (At5g55200). The Ubiquitin 5 (UB5) gene was taken as internal standard, and qRT-PCR was performed as described in Methods. Values are given as means (± SEM) of three technical replicates. This validation was repeated three times with independent biological samples, and gave similar trends. [file 1471-2164-8-450-S7.pdf]
