# Supplementary material for: Genome-wide interacting effects of sucrose and herbicide-mediated stress in Arabidopsis thaliana: novel insights into atrazine toxicity and sucrose-induced tolerance
Source: BMC Genomics. 2007 Dec 5;8:450. doi: 10.1186/1471-2164-8-450 (PMC2242805; doi:10.1186/1471-2164-8-450)
Supplement: Additional file 8 — Statistical significance of gene repression and induction for the different functional categories. The statistical analysis using a χ2 test was realized on each functional category of genes in order to compare the significance of induction and repression between the different comparisons (MA/M, S/M, SA/M). [file 1471-2164-8-450-S8.pdf]

Statistical significance of gene repression and induction for the different functional categories

Functional categories	Comparison of SA/M and MA/M		Comparison of SA/M and S/M	
	Repression	Induction	Repression	Induction
C-compound and Carbohydrate Metabolism	ns	ns	ns	$P = 0.012$
Cell Fate, Development and Biogenesis	$P = 0.040$	ns	ns	ns
Cellular Communication and Signal Transduction Mechanism	ns	$P = 0.001$	ns	$P = 0.000$
Detoxification, Degradation of Foreign (exogenous) Compounds	ns	$P = 0.008$	ns	$P = 0.002$
Disease, Virulence and Defence	ns	ns	ns	ns
DNA and RNA processing, Nucleotide Metabolism	$P = 0.005$	ns	ns	$P = 0.020$
Electron Transport and Membrane-associated energy conservation	ns	ns	ns	ns
Lipid and Fatty Acid Metabolism	ns	ns	ns	ns
Nitrogen and Sulfur Metabolism	$P = 0.014$	ns	ns	ns
Oxidative Stress Response	ns	$P = 0.000$	ns	$P = 0.002$
Plant Hormonal Regulation	ns	ns	ns	ns
Protein Degradation, Cell Aging	ns	$P = 0.013$	ns	$P = 0.013$
Protein Folding and Stabilization	$P = 0.015$	ns	ns	ns
Protein Synthesis, Protein Fate	$P = 0.000$	ns	ns	ns
Secondary Metabolism	ns	ns	ns	ns
Stress response	ns	ns	ns	$P = 0.008$
Transcription	$P = 0.042$	ns	ns	$P = 0.005$

ns : not significant, test with a χ^2 *P*-values higher than 5% were considered as being not significantly different
