# Supplementary material for: Genome-wide interacting effects of sucrose and herbicide-mediated stress in Arabidopsis thaliana: novel insights into atrazine toxicity and sucrose-induced tolerance
Source: BMC Genomics. 2007 Dec 5;8:450. doi: 10.1186/1471-2164-8-450 (PMC2242805; doi:10.1186/1471-2164-8-450)
Supplement: Additional file 9 — Repression by atrazine of genes in DNA and protein dynamics. Additional file 9 lists several genes involved in DNA and protein dynamics and repressed by atrazine treatment in the present study. [file 1471-2164-8-450-S9.pdf]

Repression by atrazine of genes involved in DNA and protein dynamics				
		log2(ratio)		
Accession		Treatment comparison		
number	Gene description	MA/M	S/M	SA/M
At1g09690	60S ribosomal protein L21 (RPL21C)	-1.93	0.75	nde
At1g26880	60S ribosomal protein L34 (RPL34A)	-2.07	1.01	nde
At1g27400	60S ribosomal protein L17 (RPL17A)	-1.85	0.74	nde
At1g64880	Ribosomal protein S5 family protein	-1.84	1.21	nde
At1g77940	60S ribosomal protein L30 (RPL30B)	-2.23	nde	nde
At2g19750	40S ribosomal protein S30 (RPS30A)	-2.04	1.07	nde
At2g21790	Ribonucleoside-diphosphate reductase small chain, putative / ribonucleotide reductase, putative	-1.80	0.87	nde
At2g32060	40S ribosomal protein S12 (RPS12C)	-1.74	1.18	nde
At2g36620	60S ribosomal protein L24 (RPL24A)	-2.38	0.77	nde
At3g04840	40S ribosomal protein S3A (RPS3aA)	-2.25	0.82	nde
At3g05590	60S ribosomal protein L18 (RPL18B)	-1.97	0.95	nde
At3g09200	60S acidic ribosomal protein P0 (RPP0B)	-1.83	nde	nde
At3g11510	40S ribosomal protein S14 (RPS14B)	-1.79	nde	nde
At3g16080	60S ribosomal protein L37 (RPL37C)	-1.99	0.90	nde
At3g23390	60S ribosomal protein L36a/L44 (RPL36aA)	-2.37	0.79	nde
At3g23830	Glycine-rich RNA-binding protein, putative	-2.13	1.05	nde
At3g48930	40S ribosomal protein S11 (RPS11A)	-1.72	1.03	nde
At3g55280	60S ribosomal protein L23A (RPL23aB)	-2.00	1.13	nde
At3g57150	Dyskerin, putative / nucleolar protein NAP57, putative	-1.75	1.03	nde
At3g60245	60S ribosomal protein L37a (RPL37aC)	-2.43	nde	nde
At3g61820	Aspartyl protease family protein	-2.44	0.75	nde
At4g09320	Nucleoside diphosphate kinase 1 (NDK1)	-1.95	0.86	nde
At4g12600	Ribosomal protein L7Ae/L30e/S12e/Gadd45 family protein	-2.48	0.99	nde
At4g25340	Immunophilin-related / FKBP-type peptidyl-prolyl cis-trans isomerase-related	-1.73	nde	nde
At4g25630	Fibrillarin 2 (FIB2)	-2.61	1.50	nde
At4g25890	60S acidic ribosomal protein P3 (RPP3A)	-1.94	0.51	nde
At4g28360	Ribosomal protein L22 family protein	-1.98	nde	nde
At4g30800	40S ribosomal protein S11 (RPS11B)	-1.88	1.50	nde
At5g02870	60S ribosomal protein L4/L1 (RPL4D)	-1.82	1.17	nde
At5g08180	Ribosomal protein L7Ae/L30e/S12e/Gadd45 family protein	-1.88	0.88	nde
At5g10390	Histone H3	-1.77	0.76	nde
At5g14580	Polyribonucleotide nucleotidyltransferase, putative	-1.82	1.46	0.86
At5g16130	40S ribosomal protein S7 (RPS7C)	-1.92	1.03	nde
At5g22440	60S ribosomal protein L10A (RPL10aC)	-1.93	1.26	nde
At5g45775	60S ribosomal protein L11 (RPL11D)	-2.00	0.95	nde
At5g56710	60S ribosomal protein L31 (RPL31C)	-1.94	0.77	nde
At5g59850	40S ribosomal protein S15A (RPS15aF)	-2.46	0.96	nde
At5g59870	Histone H2A, putative	-2.01	nde	nde
At5g60670	60S ribosomal protein L12 (RPL12C)	-2.09	0.94	nde
At5g61170	40S ribosomal protein S19 (RPS19C)	-1.72	1.08	nde

nde: not differentially expressed, genes with a Bonferroni *P*-values higher than 5% were considered as being not differentially expressed as described in Lurin *et al.* [75].
