# Supplementary material for: Genome-wide interacting effects of sucrose and herbicide-mediated stress in Arabidopsis thaliana: novel insights into atrazine toxicity and sucrose-induced tolerance
Source: BMC Genomics. 2007 Dec 5;8:450. doi: 10.1186/1471-2164-8-450 (PMC2242805; doi:10.1186/1471-2164-8-450)
Supplement: Additional file 10 — Sucrose treatment in Arabidopsis thaliana: comparison of its transcriptomic effects with previous studies. Additional file 10 lists several genes corresponding to typical markers of carbohydrate responses according to previous studies and found responsive to sucrose treatment in the present study. [file 1471-2164-8-450-S10.pdf]

Sucrose treatment in *Arabidopsis thaliana*: comparison of its transcriptomic effects with previous studies

Accession number	Gene description	Effect of sucrose	In accordance with
At5g18170	Glutamate dehydrogenase (GDH1)	repressed	Thum *et al*., 2004
At3g47340	asparagine synthetase (ASN1)	repressed	Gonzali *et al*., 2006 ; Thum *et al*., 2004
At1g03090	3-Methylcrotonyl-coenzyme A carboxylase (MCCase)	repressed	Che *et al*., 2002 ; Koch, 1996
At2g13360	Serine-glyoxylate	repressed	Koch, 1996 and Thum *et al*., 2004
At2g38400	alanine-glyoxylate	repressed	Koch, 1996 and Thum *et al*., 2004
At3g22370	alternative oxidase (AOX1A)	induced	Gonzali *et al*., 2006 ; Thum *et al*., 2004
At1g37130	nitrate reductase (NR2)	induced	Koch, 1996 ; Oswald *et al*., 2001
At3g04120	glyceraldehyde-3-phosphate dehydrogenase (GAPD)	induced	Yang *et al*., 1993
At5g41670	phosphogluconate dehydrogenase	induced	Gonzali *et al*., 2006
-	genes encoding lipases	repressed	Martin *et al*., 2002

Che P, Wurtele ES, Nikolau BJ: **Metabolic and environmental regulation of 3-methylcrotonyl-coenzyme A carboxylase expression in Arabidopsis**. *Plant Physiol* 2002, **129**:625-637.

Gonzali S, Loreti E, Solfanelli C, Novi G, Alpi A, Perata P: **Identification of sugar-modulated genes and evidence for *in vivo* sugar sensing in Arabidopsis.** *J Plant Res* 2006, **119**:115-123.

Koch KE: **Carbohydrate-modulated gene expression in plants.** *Annu Rev Plant Physiol Plant Mol Biol* 1996, **47**:509-540.

Martin T, Oswald O, Graham IA: **Arabidopsis seedling growth, storage lipid mobilization, and photosynthetic gene expression are regulated by carbon:nitrogen availability.** *Plant Physiol* 2002, **128**:472-481.

Oswald O, Martin T, Dominy PJ, Graham IA: **Plastid redox state and sugars: interactive regulators of nuclear-encoded photosynthetic gene expression.** *Proc Natl Acad Sci USA* 2001, **98**:2047-2052.

Thum KE, Shin, MJ, Palenchar PM, Kouranov A, Coruzzi GM: **Genome-wide investigation of light and carbon signaling interactions in Arabidopsis.** *Genome Biol* 2004, **5**:R10.

Yang YH, Dudoit S, Luu P, Lin DM, Peng V, Ngai J, Speed TP: **Normalization for cDNA microarray data: a robust composite method addressing single and multiple slide systematic variation.** *Nucleic Acids Res* 2002, **30**:e15.
