# Supplementary material for: Genome-wide interacting effects of sucrose and herbicide-mediated stress in Arabidopsis thaliana: novel insights into atrazine toxicity and sucrose-induced tolerance
Source: BMC Genomics. 2007 Dec 5;8:450. doi: 10.1186/1471-2164-8-450 (PMC2242805; doi:10.1186/1471-2164-8-450)
Supplement: Additional file 11 — Conserved cis-acting elements and their percentage of occurrence in the promoter region of genes presenting high induction by sucrose-atrazine combination. Additional file 11 lists cis-acting regulatory elements and their occurrence in promoters of genes presenting high induction by the sucrose-atrazine combination and corresponding transcription factors. Analysis of cis-acting regulatory elements was carried out with the AtcisDB database [file 1471-2164-8-450-S11.pdf]

Conserved cis-acting elements and their percentage of occurrence in the promoter region of genes presenting high induction by sucrose-atrazine combination

| Name of the cis-acting element | Corresponding transcription factor | Consensus sequence | Percentage of occurrence in Group V | Percentage of occurrence in the genome | *P*-value |
|---|---|---|---|---|---|
| W-box | WRKY | TTGAC | 89.47 % | 64.03 % | $P = 0.021$ |
| MYB4 binding site | MYB | A(A/C)C(A/T)A(A/C)C | 84.21 % | 48.96 % | $P = 0.002$ |
| MYB binding site | MYB | (A/C)ACC(A/T)A(A/C)C | 73.68 % | 16.71 % | $P = 0.000$ |
| DPBF1&2 binding site | bZIP | ACACXXG | 78.94 % | 41.16 % | $P = 0.001$ |
| LFY binding site | LFY | CCAXTG | 73.68 % | 43.56 % | $P = 0.008$ |
| RAV1 binding site | ABI3VP1 | CAACA | 78.94 % | 72.93 % | ns |

ns : not significant; comparisons with $\chi^2$ *P*-values higher than 5% were considered as being not significantly different
