# Supplementary material for: Genome-wide interacting effects of sucrose and herbicide-mediated stress in Arabidopsis thaliana: novel insights into atrazine toxicity and sucrose-induced tolerance
Source: BMC Genomics. 2007 Dec 5;8:450. doi: 10.1186/1471-2164-8-450 (PMC2242805; doi:10.1186/1471-2164-8-450)
Supplement: Additional file 12 — Genes selected for qRT-PCR analysis and primer sequences. Additional file 12 lists genes selected for qRT-PCR analysis and primer sequences. [file 1471-2164-8-450-S12.pdf]

Genes selected for qRT-PCR analysis and primer sequences			
Accession number	Gene description	Forward sequence	Reverse sequence
At1g06570	4-hydroxyphenylpyruvate dioxygenase (PDS1)	TCGCTCGTCGCTTCTCCTG	TGTGGTTGTCGGTTTAATCTCTCC
At1g42990	bZIP transcription factor family protein	TCTGCTGTGCTCTTGTTGGAATC	GAACCCTTACATCTCCGACTAACG
At1g75270	Glutathione dehydrogenase (ascorbate)	ACAAGAACTGGTCTGTCCCTGAG	CCACATCACGCATTCACCTTCG
At1g77210	Carbohydrate transporter / sugar porter	GCATCCTCGTTGGCTCTG	ATTCACAGTTCCTCTAATCTTCG
At2g34500	CYP710A1	GACCTCCCTGGCTTTGCGTTCG	GTGCGGTGGCTGCGGATTCTC
At2g47000	ATPase	GGAAGCAAGCCAAGTAGCGAATG	GACGAAGAAAGAGAAGCCGAAACC
At3g09270	Glutathione transferase	TGGAAGACAACTCACACG	ACATTTCAAACCCTCATAAGC
At3g56710	sigA-binding protein	CCTTCCTCCGACGACCACAAC	GAACGGCTCCTGATGAAGAACAC
At3g61630	AP2 domain-containing transcription factor	ACAGAGGCGTGAGACAGAGAC	TCGGCGGAGTTAGGAAATTCG
At3g62250	Ubiquitin 5 (UBQ5)	CCAAGCCGAAGAAGATCAAG	TCAAAATGACTCGCCATGAA
At5g40760	Glucose-6-phosphate 1-dehydrogenase (G6PD6)	AACGGTATCAAGGTGTCGCTATCC	CCTCGGCTTCCTGGTTTGTATGG
At5g44340	tubulin beta-4 chain (TUB4)	ACCTTATCCCATTCCCAAGG	CAGCGGATGCAGTCAAGTAA
At5g55200	Chaperone GrpE-like protein	CTCCTCCGTCGTTACTCC	TGTTACTCTCATTTCCCTTCG
At5g59820	zinc finger family protein (ZAT12)	GATGTCACGGCGGCGAATTG	AGGCTTGGAACGAATGAAACTGC
